# Endoplasmic reticulum stress in non-small cell lung cancer: a review of therapeutic agents, mechanistic insights, and implications for therapy

**DOI:** 10.3389/fcell.2025.1693023

**Published:** 2025-12-16

**Authors:** Qiong Luo, Xinxin Gao, Peng Meng, Xiao Qi, Wen Wang, Shan Li, Linlin Duan

**Affiliations:** 1 Department of Respiratory and Critical Care Medicine, Affiliated Hospital Group of Guangdong Medical University Shenzhen Baoan Central Hospital (Baoan Central Hospital of Shenzhen), Shenzhen, Guangdong, China; 2 Department of Thoracic Surgery, Yantai Hospital of Traditional Chinese Medicine, Yantai, Shandong, China; 3 Department of Oncology, Yantai Hospital of Traditional Chinese Medicine, Yantai, Shandong, China; 4 Department of Pulmonary Diseases, Yantai Hospital of Traditional Chinese Medicine, Yantai, Shandong, China; 5 Department of Blood disease, Yantai Hospital of Traditional Chinese Medicine, Yantai, Shandong, China

**Keywords:** non-small cell lung cancer, endoplasmic reticulum stress, unfolded protein response, therapy resistance, immunogenic cell death

## Abstract

Non-small-cell lung cancer (NSCLC) remains a leading cause of cancer-related mortality, with therapy resistance significantly hindering treatment efficacy. This review explores the role of endoplasmic reticulum (ER) stress and the unfolded protein response (UPR) in NSCLC progression and resistance mechanisms. Under stress conditions such as hypoxia, nutrient deprivation, or therapeutic insult, the UPR balances adaptive survival signaling and apoptotic pathways. Key UPR sensors—PERK, IRE1α, and ATF6—are dysregulated in NSCLC, enabling tumor cells to evade death despite microenvironmental or treatment-induced stress. Preclinical studies highlight therapeutic strategies targeting ER stress through reactive oxygen species (ROS) induction, calcium homeostasis disruption, and proteasome inhibition, which shift the UPR toward pro-apoptotic outcomes. Agents such as proteasome inhibitors, natural compounds, and repurposed drugs demonstrate the potential to overcome resistance by enhancing chemosensitivity, reversing chemoresistance, and improving radiosensitivity. Combination therapies synergize ER stress inducers with conventional treatments, leveraging immunogenic cell death (ICD) to augment anti-tumor immunity. However, challenges persist due to the UPR’s context-dependent outputs and the gap between preclinical models and clinical applicability. Future directions include optimizing combination regimens, identifying predictive biomarkers, and advancing personalized approaches. Translating these insights into clinical trials is critical to validate ER stress modulation as a viable strategy for improving NSCLC outcomes, offering a promising avenue to address unmet needs in this aggressive malignancy.

## Graphical Abstract

**Figure:**
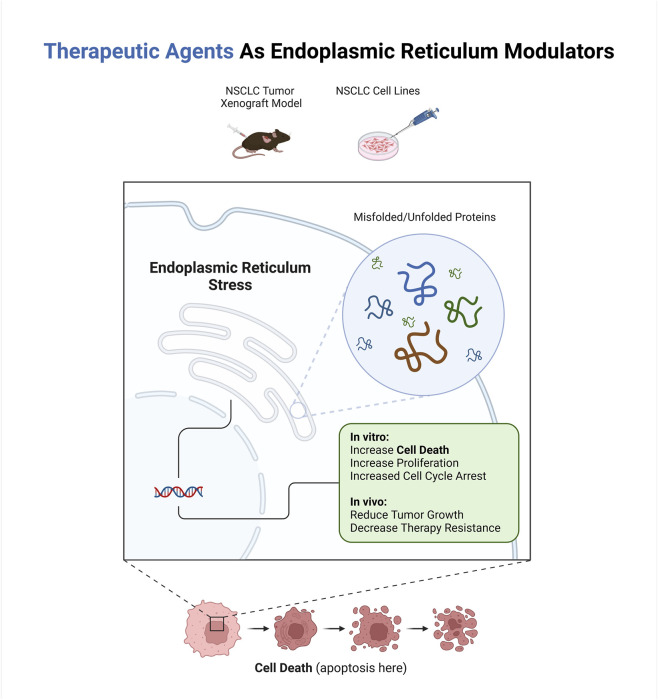

## Highlights

• ER stress in NSCLC balances pro-survival signaling and apoptosis induction.
• Targeting PERK/CHOP and IRE1/JNK pathways enhances NSCLC therapy efficacy.
• ER stress inducers overcome chemotherapy and radiotherapy resistance.
• Calcium dysregulation and ROS amplify ER stress-driven tumor cell death.
• ER stress triggers immunogenic cell death, boosting anti-tumor immunity.

## 1 Introduction

Non-small-cell lung cancer (NSCLC) represents the majority of all lung cancer cases globally, consisting of three primary histological subtypes: lung adenocarcinoma (LUAD), which originates from mucus-producing gland cells; lung squamous cell carcinoma (LSCC), derived from the squamous epithelium of the airways; and lung large cell carcinoma (LLCC), an undifferentiated group of epithelial tumors (Hendriks et al., 2024). In 2024, an estimated 2,001,140 new cancer cases were expected in the United States, with NSCLC playing a key role in cancer-related deaths (Siegel et al., 2024; Ergun, 2024; Wen et al., 2023). The 5-year survival rate remains poor, with only 8% for metastatic cases. However, progress in immunotherapy and targeted treatments has led to modest improvements in outcomes for patients with specific biomarkers (Hendriks et al., 2024; Song et al., 2024).

There are different therapies for NSCLC, including targeted therapy, surgery, radiation, chemotherapy, and immunotherapy, each with benefits and limitations. Targeted therapies improve survival by addressing mutations like EGFR, ALK, ROS1, and KRAS but face resistance, bypass signaling, and tumor heterogeneity. Surgery is effective for early stages but has high recurrence risks, often requiring additional therapy. Radiation and chemotherapy are key treatments but may develop therapy resistance and significant toxicity that have limited long-term benefits. Immunotherapy enhances the immune response but is only effective in some patients and can cause immune-related side effects (Alduais et al., 2023; Wu and Lin, 2022; Cheng et al., 2024; Lalić et al., 2024; Wu Z. et al., 2024).

Despite ongoing research in NSCLC treatment, therapy resistance remains a significant challenge, often driven by cellular adaptations that enable tumor survival under adverse conditions. Among these mechanisms, endoplasmic reticulum (ER) stress is pivotal in promoting tumor progression and resistance to therapy (Qian et al., 2024; Liu et al., 2023; Wang Y. et al., 2023; Wang N. et al., 2023).

ER stress is a condition that arises within cells when the ER’s capacity to fold proteins properly becomes overwhelmed. Cancer cells frequently undergo ER stress due to internal factors like oncogene activation and external stressors such as drugs. These stressors disrupt the normal proteostasis process, triggering a series of cellular responses collectively known as the unfolded protein response (UPR). The UPR aims to restore normal function by reducing protein synthesis, enhancing protein folding, and promoting degradation of misfolded proteins (Chen et al., 2023). Research has shown that ER stress and UPR are highly induced in various tumors and closely linked to cancer cell survival, making them potential therapeutic targets (Marfatiya et al., 2025). For instance, sustained ER stress can trigger apoptosis, offering a mechanism to target cancer cells selectively (Wu et al., 2020).

However, the adaptive responses of the UPR can also support tumor growth by mitigating stress and enabling cancer cells to thrive under adverse conditions (Chevet et al., 2015). Moreover, the interplay between oncogenic signaling and ER stress response further complicates this relationship, as ER stress signaling is dysregulated in many cancers and contributes to tumor growth (Chen and Cubillos-Ruiz, 2021). This dysregulation highlights the vulnerability of cancer cells to targeted interventions against ongoing UPR pathways (Oakes, 2020). Additionally, studies have clarified the link between ER stress and specific cancer types, such as renal cell carcinoma, where ER stress activation or suppression influences cancer progression (Corr et al., 2023). Therefore, understanding the balance between adaptive and apoptotic roles of ER stress in cancer could provide insights into developing novel therapeutic strategies to exploit these vulnerabilities (Yadav et al., 2014).

Herein, we aim to synthesize current knowledge on the dual role of ER stress and the UPR in NSCLC progression and therapy resistance, focusing on evaluating recent therapeutic strategies that target these pathways. By analyzing emerging evidence on ER stress mechanisms—including its adaptive pro-survival signaling and potential to induce apoptosis under sustained stress—this review seeks to elucidate how modulating these pathways could overcome resistance to conventional therapies and improve treatment outcomes.

## 2 An overview of ER stress molecular mechanisms

The ER is a critical organelle in eukaryotic cells responsible for protein synthesis, folding, post-translational modifications, and lipid biosynthesis. Proper protein folding is essential for cellular function, and the ER maintains a highly regulated environment to ensure this process occurs efficiently (Schwarz and Blower, 2016). Unlike DNA replication, protein folding is notably error-prone. Therefore, preserving a functional proteome relies on complex quality control mechanisms, some of which function within the ER to facilitate proper protein folding and trafficking (Adams et al., 2019).

### 2.1 The UPR pathways

Under optimal conditions, the ER ensures proteins are correctly folded into functional conformations. However, an imbalance between the load of unfolded/misfolded proteins and the capacity of the ER to handle them happens when cells are under internal (oncogenes activation, hypoxia, nutrient deprivation) (Almanza et al., 2019; Dabsan et al., 2025; Ghemrawi et al., 2025) or external (chemotherapy, radiotherapy, or other therapies disrupting protein folding, oxidative stresses, infections) stresses (Avril et al., 2017; Xiong et al., 2021; Gardner et al., 2013). Thus, the capacity of the ER to fold proteins becomes overwhelmed, leading to an accumulation of unfolded/misfolded proteins within the ER lumen (Chen et al., 2023).

These conditions disrupt proteostasis (protein homeostasis) and trigger a series of double-edged responses collectively known as the UPR. The primary objective of the UPR is to restore ER homeostasis and ensure cell survival via reprogramming gene transcription, mRNA translation, and post-translational protein modifications to alleviate the misfolded protein burden (Hetz et al., 2020).

The UPR pathways play a dual role in regulating cell fate. Under mild or transient stress, it triggers *adaptive* processes that restore ER balance and support cell survival. However, when ER stress becomes excessive or persists beyond a critical threshold, the UPR transitions to a *pro-apoptotic* state (Chowdhury et al., 2024).

**FIGURE 1:**
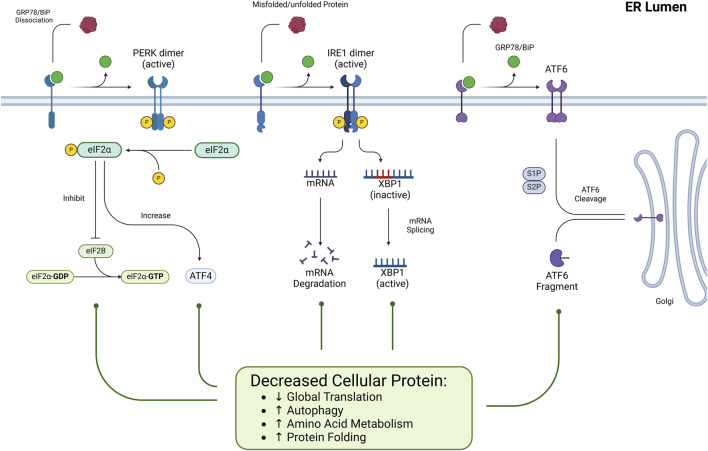
*The role of ER stress and the UPR pathways under normal conditions*. Under normal physiological conditions, ER stress is minimal, and the Unfolded UPR maintains cellular homeostasis. ER-resident sensors PERK, IRE1, and ATF6 remain inactive through their association with the chaperone GRP78, thereby preventing unnecessary activation of stress responses. This inactive state ensures efficient ER protein folding, translation, and degradation processes, preserving proteostasis and supporting cell survival.

**FIGURE 2:**
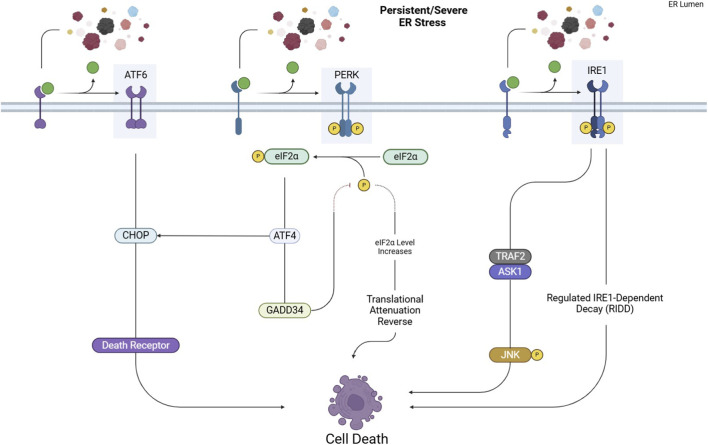
*UPR pathways under prolonged ER stress*. Excessive or persistent ER stress shifts the UPR from adaptive to apoptotic responses. Activated PERK, IRE1, and ATF6 trigger pro-apoptotic signaling through key effectors like CHOP and JNK, ultimately leading to cell death.

This pathway consists of three main axes, each initiated by distinct ER transmembrane sensors: inositol-requiring enzyme 1 (IRE1), PKR-like ER kinase (PERK), and activating transcription factor 6 (ATF6). Figures 1, 2 represent the cell fate in normal conditions and under prolonged ER stress, respectively.

The shift from an adaptive to a pro-apoptotic UPR is not random but depends on the intensity and duration of stress along with multiple regulatory checkpoints. Recent studies show that individual UPR branches possess distinct activation thresholds that determine cell fate. In the PERK–ATF4 axis, graded phosphorylation of eIF2α allows initial survival responses (antioxidant signaling, amino acid metabolism), but sustained activation exceeds a threshold that drives CHOP-mediated apoptosis, partially through transcriptional induction of BIM, PUMA, and death receptor 5 (DR5) (Hetz and Papa, 2018). In parallel, PERK promotes adaptive outcomes via NRF2, while its prolonged activity reverses translational attenuation through GADD34, worsening proteotoxic overload and pushing the system toward cell death. Similarly, IRE1α integrates temporal stress signals through modulation of its kinase/RNase activities. Under moderate stress, IRE1 primarily favors XBP1s-driven survival, upregulating ERAD and lipid biosynthesis. With persistent stress, however, conformational oligomerization converts IRE1α into a hyperactive nuclease, enabling RIDD and activation of ASK1–JNK signaling—both promoting apoptosis (Hetz and Papa, 2018; Jaud et al., 2020). These divergent outputs are further regulated by post-translational modifications (e.g., phosphorylation, ubiquitination, ADP-ribosylation) and regulatory cofactors (e.g., BCL-2 family proteins, PDIA6, BI-1), which collectively form the dynamic *UPRosome* molecular platform that dictates cell fate. In cancer, additional context-dependent factors, including hypoxia, metabolic reprogramming, oncogenic signaling, and tumor microenvironment, modulate these UPR fate checkpoints. In NSCLC, this mechanistic ambiguity allows tumor cells to exploit UPR survival signaling for immune evasion and therapeutic resistance, while chronic stress or pharmacologic UPR hyperactivation can instead drive apoptotic elimination.

#### 2.1.1 PERK pathway

Under normal conditions (Figure 1), PERK is kept inactive by its association with the binding immunoglobulin protein (BiP or GRP78) chaperone that helps proteins fold correctly. When unfolded/misfolded proteins accumulate in the ER lumen, GRP78 dissociates from PERK because it preferentially binds to these misfolded proteins to assist in their refolding. As a result, PERK is dimerized and undergoes autophosphorylation, which fully activates its kinase activity. Activated PERK phosphorylates the eukaryotic translation initiation factor 2 alpha (eIF2α) (He et al., 2024).

Phosphorylated eIF2α (p-eIF2α) inhibits the formation of the eIF2-GTP-tRNA complex, reducing overall global protein synthesis. p-eIF2α inhibits global protein synthesis by sequestering eIF2B, preventing eIF2-GDP from being reactivated to eIF2-GTP, and blocking the formation of the ternary complex needed for translation initiation (Bellato and Hajj, 2016). This reduction in translation is a protective strategy to lessen the influx of newly synthesized proteins into the already overloaded ER (Liu et al., 2024). This mechanism is not absolute and Activating Transcription Factor 4 (ATF4) mRNAs can bypass the global translation blockade. ATF4 then moves to the nucleus, activating genes related to amino acid metabolism, chaperone molecules, and autophagy (Yang et al., 2024).

However, if the cellular stress is severe or prolonged (Figure 2), ATF4 triggers pro-apoptotic signals by activating downstream effectors such as C/EBP homologous protein (CHOP) (Rozpedek et al., 2016).

CHOP promotes apoptosis through multiple mechanisms, including upregulating the expression of Growth Arrest and DNA Damage-Inducible Protein 34 (GADD34), which dephosphorylates eIF2α, reversing the translational attenuation initially induced by PERK. This can lead to an overload of unfolded proteins, exacerbating ER stress and pushing the cell toward apoptosis (Rozpedek et al., 2016).

Also, CHOP induces apoptosis via the death receptor pathway by upregulating death receptors like DR4 and DR5. CHOP regulates DR4 and DR5 expression in different cell types. ATF3 also contributes to DR5 production. Persistent ER stress leads to the accumulation of DR5, which promotes the multimerization of DR5 ligands (TRAIL, TNF, and Fas), accelerating the formation of the death-inducing signaling complex (DISC) and activating caspase-8. Caspase-8 activation triggers apoptosis through both extrinsic and intrinsic pathways by cleaving BID into tBID, which interacts with BAX and BAK to release cytochrome c, ultimately leading to cell death (Hu et al., 2018).

#### 2.1.2 IRE1 pathway

Under normal conditions (Figure 1), IRE1 is kept in an inactive monomeric state through its association with BiP. However, when ER stress occurs, the IRE1 transmembrane protein structure detects ER stress via its luminal domain. Then, BiP detaches from IRE1 to interact with misfolded proteins, triggering IRE1 dimerization and oligomerization, which subsequently activates its kinase domain via autophosphorylation. This phosphorylation further activates the RNase domain (Cho et al., 2019).

The RNase domain of activated IRE1 removes a 26-nucleotide intron from XBP1 mRNA, producing the spliced variant (XBP1s). XBP1s enhance the expression of genes responsible for protein folding, ER-associated degradation (ERAD), and other mechanisms that mitigate ER stress (Siwecka et al., 2021).

Besides splicing XBP1, activated IRE1 can also degrade specific mRNAs and non-coding RNAs associated with the ER membrane via the regulated IRE1-dependent decay (RIDD) process. It helps decrease the influx of newly synthesized proteins into the ER, thereby reducing ER stress (Chen et al., 2023).

However, when cellular stress remains severe or persistent (Figure 2), IRE1 initiates pro-apoptotic signaling by activating downstream effectors. IRE1 recruits TNF Receptor-Associated Factor 2 (TRAF2), activating Apoptosis Signal-regulating Kinase 1 (ASK1). This leads to the phosphorylation and activation of c-Jun N-terminal kinase (JNK), a type of protein kinase involved in cellular signaling. It is part of the Mitogen-Activated Protein Kinase (MAPK). Once activated, JNK moves to the mitochondrial membrane, phosphorylating and inhibiting anti-apoptotic Bcl-2 family proteins, including Bcl-2 and Bcl-xL. This inhibition results in cytochrome c release and the activation of the intrinsic apoptotic pathway (Junjappa et al., 2018).

Although the RIDD pathway reduces the load of newly synthesized proteins in the ER, it also leads to the degradation of critical ER resident proteins. The loss of these essential proteins can trigger apoptosis, as it disrupts normal ER function and homeostasis. Additionally, RIDD can de-repress Caspase 2, further promoting apoptotic pathways (Coelho and Domingos, 2014).

#### 2.1.3 ATF6 pathway

ATF6 is maintained inactive by binding with the chaperone protein GRP78 without stress (Figure 1) (Yuan et al., 2024). Under ER stress, BiP dissociates from ATF6, allowing ATF6 to exit its inactive state. Then, ATF6 is transported to the Golgi apparatus, where it undergoes sequential proteolytic cleavage by Site-1 Protease (S1P) and Site-2 Protease (S2P). This cleavage releases the N-terminal cytoplasmic domain of ATF6, which functions as a transcription factor. The active fragment translocates to the nucleus, upregulating genes involved in the ER stress response, including molecular chaperones, protein folding enzymes, and ERAD components (Allen and Seo, 2018).

However, ATF6 induces apoptosis by upregulating CHOP under persistent stress, which disrupts cellular balance and triggers apoptotic signaling. This process is also regulated by factors like GRP78/BiP and β-arrestin2 and can be further influenced by P53 and inflammatory pathways (Yang et al., 2020).

### 2.2 ER stress and non-apoptotic cell death

Paraptosis is a type of programmed cell death that differs from apoptosis and necrosis. This process is characterized by forming vacuoles in the cytoplasm and the enlargement of cellular structures like the mitochondria and ER. Notably, it does not exhibit key apoptotic traits, such as DNA fragmentation. Since paraptosis occurs without activating caspases, it provides an alternative approach for eradicating cancer cells that no longer respond to standard apoptosis-triggering therapies (Hanson et al., 2023; Xu CC. et al., 2024).

A key mechanism during ER stress involves excessive calcium (Ca^2+^) release from the ER into the cytosol, leading to mitochondrial overload, dysfunction, and swelling—hallmark features of paraptosis. The interplay between the ER and mitochondria via Ca^2+^ flux is crucial for the progression of this cell death pathway (Kunst et al., 2024). In cancer cells under ER stress, inhibiting or deleting the PERK pathway triggers the activation of SEC61β, a component of the ER translocon. This activation leads to SEC61β-driven paraptosis and promotes immunogenic cell death (ICD) (Mandula et al., 2022).

ICD is a regulated cell death that triggers an immune response by releasing damage-associated molecular patterns (DAMPs), such as calreticulin exposure, ATP secretion, and high mobility group box 1 (HMGB1) release. These signals activate the immune system, promoting innate and adaptive immunity against antigens from dying cells (Rodrigues et al., 2022). ICD enhances tumor immunogenicity in cancer biology, making it a key factor in immunotherapy by shaping prognosis and treatment strategies. Its ability to induce long-term immunological memory supports sustained anti-tumor effects (Arimoto et al., 2024).

Additionally, paraptosis induction has been linked to the activation of MAPK/JNK signaling pathways. These pathways are activated downstream of receptors such as IGF-IR (insulin-like growth factor receptor-I), which mediates transcriptional and translational processes required for paraptosis. The activation of these kinases contributes to cellular stress responses, including ER overload and oxidative stress, which further drive the paraptosis process (Chang et al., 2024).

## 3 Mechanisms of ER stress modulation in NSCLC

In this section, we have provided a comprehensive overview of therapeutic agents targeting ER stress in the pathogenesis of NSCLC. Research indicates numerous drugs influence ER function and protein homeostasis through diverse pathways. Here, we have highlighted how various therapeutic agents leverage ER stress and the UPR pathways to exert their therapeutic effects.

### 3.1 ER stress induction mechanisms

Several mechanisms contribute to ER stress, including oxidative stress, calcium dysregulation, and protein misfolding, which disrupt ER homeostasis and activate the UPR. Key ER stress sensors—PERK, IRE1α, and ATF6—mediate downstream signaling pathways influencing cell survival. Therefore, understanding how different agents trigger ER stress through these mechanisms provides insight into their therapeutic potential in NSCLC treatment.

The common ER stress triggers are deeply interconnected, often influencing one another in a complex feedback loop that exacerbates cellular stress.

Reactive oxygen species (ROS) serve as a primary driver of ER stress by disrupting ER homeostasis, leading to the activation of the UPR. This disruption is closely tied to calcium dysregulation, as ROS can impair ER calcium handling, contributing to ER Ca^2+^ depletion and mitochondrial Ca^2+^ overload (Cao and Kaufman, 2014).

Calcium dysregulation, in turn, exacerbates protein misfolding, as ER Ca^2+^ is crucial for proper protein folding. When Ca^2+^ levels are disrupted, chaperone proteins lose their function, accumulating misfolded proteins. Additionally, proteasome inhibition or Aggresome disruption compounds the issue by preventing the degradation of misfolded proteins, further amplifying ER stress (Lee and Song, 2021). Similarly, cholesterol and calcium signaling are closely linked. ER cholesterol depletion triggers Reticulophagy (ER-specific autophagy) and stress responses. Cholesterol imbalances can also affect membrane integrity and Ca^2+^ homeostasis, further contributing to ER dysfunction and ROS production (Casas and Dickson, 2024).

#### 3.1.1 ROS production

ROS contribute to ER stress by disrupting ER homeostasis through protein folding disruption and calcium dysregulation. ROS (superoxide anion (O_2_^−^), hydrogen peroxide (H_2_O_2_), hydroxyl radical (•OH)) can oxidize thiol groups in proteins, leading to improper disulfide bond formation and the accumulation of misfolded proteins. Additionally, ROS impairs calcium handling by promoting calcium leakage from the ER into the cytosol and impairing calcium uptake into the ER, disrupting calcium-dependent activities necessary for proper protein folding. This calcium imbalance can also overload mitochondria, increasing mitochondrial ROS production and further exacerbating oxidative stress, creating a vicious cycle that amplifies ER stress (Cao and Kaufman, 2014; Zeeshan et al., 2016; Wang L. et al., 2024).

**FIGURE 3:**
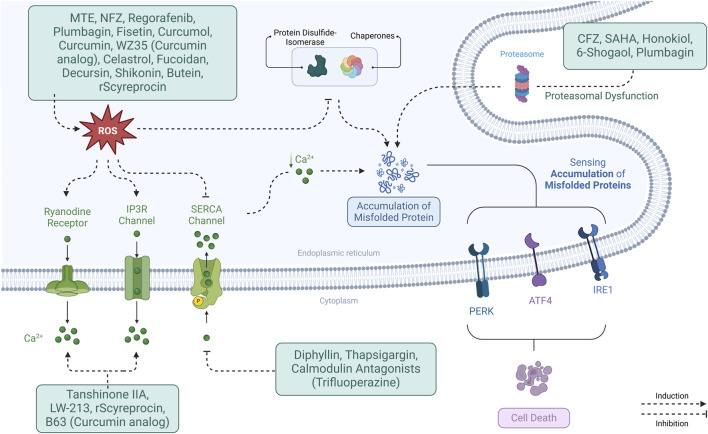
*Mechanisms of ER stress modulation in NSCLC*. ER stress is induced through interconnected pathways involving ROS, calcium dysregulation, protein misfolding, proteasome inhibition, and cholesterol imbalance. These triggers activate UPR sensors (PERK, IRE1α, ATF6), influencing cell fate and therapeutic responses.

Various therapeutic strategies have been developed to target ROS-induced ER stress (Figure 3), potentially benefiting NSCLC treatment. Several compounds have been identified that induce ROS production, thereby triggering ER stress in NSCLC cells. These agents are summarized in Supplementary Table S1.

For example, Marsdenia tenacissima extract (MTE), Nifuroxazide, Regorafenib, and Cisplatin all generate ROS, activating pathways like PERK/eIF2α/ATF4, which upregulate CHOP and trigger cell death. Other compounds Plumbagin and Fisetin induce ROS and activate similar pathways, enhancing apoptosis (Yuan et al., 2023; Li et al., 2023; Sui et al., 2023). In addition, drugs such as Curcumol (Zhang J. et al., 2020) and Celastrol (Dai et al., 2021; Zhao et al., 2022) promote ROS production and ER stress, while targeting specific proteins like GRP78 and ATF6.

Proteasome inhibitors like Carfilzomib and histone deacetylase 6 (HDAC6) inhibitors like Suberanilohydroxamic Acid increase ER stress by promoting misfolded protein accumulation. Combining these drugs with treatments like Cisplatin or TRAIL enhances their apoptotic effects (Hanke et al., 2016).

Furthermore, compounds like Shikonin (Hu et al., 2020; Li et al., 2018), Curcumin (Wang et al., 2019; Wu et al., 2010), WZ35 (a Curcumin analogue) (Dai et al., 2018) and Butein (Di et al., 2019) increase ROS, activate ER stress pathways, and induce mitochondrial dysfunction, making them promising candidates for combination therapies. Fucoidan (Hsu et al., 2017) and Decursin (Kim J. et al., 2016) also induce ROS, activate ER stress, and enhance cell death sensitivity, showing potential in synergistic cancer therapies.

In summary, a diverse range of compounds targeting ROS-induced ER stress in NSCLC cells have been identified, each through distinct mechanisms such as ROS generation, activation of key ER stress proteins like GRP78, ATF4, and CHOP, and modulation of apoptosis pathways. These agents induce mitochondrial dysfunction and cell cycle arrest, further enhancing their cytotoxic effects. Combining these agents with other therapies, such as chemotherapy or targeted therapies, holds significant potential for overcoming therapeutic resistance and improving treatment outcomes in NSCLC.

#### 3.1.2 Calcium dysregulation

Calcium imbalance is a key factor in the onset and intensification of ER stress. The connection between calcium dysregulation and ER stress is reciprocal—disruptions in ER Ca^2+^ homeostasis can initiate ER stress, while ER stress, in turn, worsens calcium imbalances. Supplementary Table S2 summarizes the therapeutic agents modulating the calcium metabolism.

The depletion of ER Ca^2+^ disrupts protein folding and chaperone activity, leading to ER stress. The ER depends on calcium to maintain the proper function of chaperones such as calreticulin and calnexin. When ER Ca^2+^ levels drop, these chaperones lose efficiency, accumulating misfolded proteins and triggering the UPR. Furthermore, ER Ca^2+^ ATPase (SERCA) inhibitors can deplete ER calcium and strongly activate ER stress pathways. These pathways attempt to restore homeostasis but may ultimately lead to cell death if stress persists (Zhao et al., 2023; Pontisso et al., 2024; Zhou et al., 2025).

Mitochondrial Ca^2+^ overload is another consequence of ER calcium dysregulation, leading to further cellular dysfunction. The ER and mitochondria are closely connected, facilitating calcium transfer between them. When ER-mitochondrial communication is excessive, mitochondria take up too much Ca^2+^, impairing their function. This results in reduced ATP production and increased ROS generation, which, in turn, disrupts ER calcium regulation, creating a cycle of oxidative stress and ER stress. Furthermore, mitochondrial Ca^2+^ overload triggers the release of apoptotic factors like cytochrome c, promoting cell death. This mitochondrial dysfunction contributes to ROS accumulation, worsening ER stress and calcium imbalance (Li et al., 2024; Mustaly-Kalimi et al., 2025).Calcium dysregulation and ER stress have a bidirectional relationship, reinforcing each other in a self-perpetuating cycle. ER stress can lead to calcium dysregulation by disrupting ER calcium channels and pumps, such as inhibiting SERCA, which decreases ER Ca^2+^ uptake. Conversely, calcium dysregulation, whether from ER Ca^2+^ depletion or mitochondrial overload, intensifies ER stress by impairing protein folding, increasing ROS levels, and activating pro-apoptotic pathways. This interconnected feedback loop makes calcium homeostasis crucial for preventing cellular dysfunction and apoptosis (Zhao et al., 2023; Sun et al., 2025). The interplay between calcium homeostasis and ER stress underscores their role in cellular fate. Building on this foundation, various pharmacological agents exploit this relationship by targeting calcium regulation and ER stress pathways to induce apoptosis alone or in combination with conventional therapies (Figure 3).

Diphyllin disrupts calcium homeostasis by inhibiting SERCA2, leading to ER Ca^2+^ depletion and subsequent ER stress. This activates key stress markers inducing apoptosis. Additionally, SERCA2 inhibition causes mitochondrial Ca^2+^ overload, triggering cytochrome c release and caspase activation, further promoting apoptosis. When combined with Cisplatin, which induces DNA damage-driven apoptosis, Diphyllin enhances Cisplatin’s cytotoxic effects by amplifying ER stress-induced cell death (Xu Z. et al., 2024).

Nifuroxazide (Li et al., 2023) and Plumbagin (Jiang et al., 2021) both increase ROS levels, leading to elevated intracellular Ca^2+^ and the activation of ER stress pathways. Similarly, Tanshinone IIA elevates Ca^2+^ levels, activating ER stress and JNK signaling while downregulating NFAT2 and c-Myc, which are associated with cellular survival (Zhang YZ. et al., 2024). LW-213, a derivative of Wogonin, inhibits NPC1, leading to cholesterol depletion in the ER. This triggers Ca^2+^ release and activation of FAM134B, promoting Reticulophagy, ER stress, and apoptosis (Wang H. et al., 2024). Recombinant scyreprocin (rScyreprocin) also increases ROS levels, which can lead to ER stress, Ca^2+^ release, mitochondrial dysfunction, and apoptosis (Yang et al., 2022).

The combination of Paclitaxel and Honokiol disrupts proteasomal function, leading to ER stress, UPR activation, and ER dilation, which induces paraptosis. This combination also disrupts Ca^2+^ homeostasis, resulting in mitochondrial Ca^2+^ overload and dysfunction, further promoting paraptosis (Li et al., 2021).

Evodiamine elevates ROS and intracellular Ca^2+^, leading to ER stress, caspase-12 activation, and apoptosis. Additionally, Evodiamine upregulates Bax while downregulating Bcl-2, promoting intrinsic apoptotic pathways via caspase-9 and caspase-3 activation (Fang et al., 2014).

Thapsigargin inhibits SERCA, depleting ER Ca^2+^ and inducing ER stress. Calmodulin antagonists like Trifluoperazine and Ophiobolin A cause ER Ca^2+^ leakage, exacerbating ER stress. When combined, thapsigargin and calmodulin antagonists lead to even stronger ER stress responses (Linxweiler et al., 2013).

Curcumin increases ROS levels, upregulating ER stress proteins such as GADD153 and GRP78, leading to apoptosis. Additionally, Curcumin promotes ER Ca^2+^ release, causing mitochondrial damage through loss of membrane potential (ΔΨm), further driving cell death (Wu et al., 2010). B63, a synthetic Curcumin analog, induces ER Ca^2+^ depletion, leading to UPR activation and apoptosis via caspase-3 and caspase-9 activation, ultimately inhibiting tumor growth (Xiao et al., 2012).

Pharmacological agents targeting calcium regulation pathways exploit calcium imbalance to enhance ER stress responses (Figure 3), leading to cancer cell death. By modulating ER Ca^2+^ levels, inducing oxidative stress, or disrupting proteostasis, these compounds demonstrate potential for therapeutic intervention, alone or in combination with existing treatments.

#### 3.1.3 Protein misfolding

Proteasome inhibition is strongly associated with ER stress due to its role in disrupting proteostasis. The proteasome is a key component of the ubiquitin-proteasome system, which degrades misfolded or damaged proteins. When proteasome function is impaired, these proteins accumulate in the ER, triggering ER stress and activating the UPR. One major consequence of proteasome inhibition is the accumulation of misfolded proteins. When proteasome function is blocked, misfolded or ubiquitinated proteins build up beyond the ER’s capacity to manage them. This overload activates ER stress pathways, leading to cellular dysfunction (Ri, 2016). Also, proteasome inhibitors sensitize cells to ER stress-induced apoptosis. This sensitization occurs through pathways not entirely dependent on ROS, indicating a direct link between proteasome inhibition and ER stress-induced cell death (Amanso et al., 2011). Supplementary Table S2 summarizes the therapeutic agents disrupting the proteasome function.

ROS-induced ER stress is pivotal in cancer cell apoptosis and has become a promising therapeutic target in NSCLC. Many compounds induce ER stress by disrupting protein homeostasis. The Carfilzomib inhibits the proteasome, accumulating misfolded proteins, while Suberanilohydroxamic Acid blocks Aggresome formation, further exacerbating ER stress. Their combination amplifies ROS production and ER stress, triggering apoptosis (Hanke et al., 2016).

Similarly, Honokiol and Paclitaxel synergistically disrupt proteasomal function, triggering ER dilation and UPR, leading to paraptosis. This effect is further enhanced by disrupting Ca^2+^ homeostasis, inducing mitochondrial dysfunction and cell death (Li et al., 2021).

Plumbagin also inhibits the proteasome and increases ER stress through sulfhydryl disruption, leading to cytoplasmic vacuolation and paraptosis (Binoy et al., 2019).

Additionally, 6-Shogaol, a ginger-derived compound, causes proteasome inhibition, polyubiquitinated protein accumulation, and ER-derived vacuolation, inducing caspase-independent paraptosis (Nedungadi et al., 2018).

Overall, targeting proteasomal dysfunction represents a compelling strategy in NSCLC treatment. These agents disrupt proteostasis, induce pro–apoptotic ER stress, and, in some cases, promote paraptotic cell death, offering alternative pathways to overcome apoptosis resistance in cancer cells.

### 3.2 Direct ER stress activators via UPR pathways

In our exploration of therapeutic agents that modulate ER homeostasis, it becomes evident that many of these compounds exert their anticancer effects by directly targeting the three main UPR branches—PERK, IRE1, and ATF6. This shifts the balance from adaptive survival to cell death. Table 1 summarizes these therapeutic agents, detailing their mechanisms of action and ultimate effects.

**TABLE 1:** *Therapeutic agents directly targeting ER stress pathway regulators*.

Therapeutic agent	Drug category	Axis	Outcomes	References
Thrombospondin-1 mimic peptide (PKHB1)	Experimental	PKHB1 → ↑GRP78, PERK, p-eIF2α, ATF4, CHOP, IRE1α, p-JNK → Apoptosis	*In Vitro*: Inhibits cell viability, proliferation, and migration, induced apoptosis via mitochondrial dysfunction and calcium overloading, and triggered ER stress to promote cell death via a CD47-independent pathway. *In Vivo*: Suppressed tumor growth and weight in xenograft models, activating ER stress and apoptosis in the tumor tissues	Ye et al. (2023)
Evodiamine (EVO)	Experimental	EVO → ↑GRP78, ↑IRE1 → IRE1/TRAF2/ASK1 complex → ↑p-JNK → ↑ Apoptosis	*In Vitro*: Inhibits cell viability and induced apoptosis, increasing the expression of apoptosis-related proteins, while promoting ERS via upregulation of GRP78 and IRE1. *In Vivo*: Reduced tumor weight and growth in LLC mice, enhanced apoptosis markers	Li et al. (2022)
Pterostilbene (PT)	Experimental	PT → ↑p-PERK → ↑IRE1 → ↑ATF4 → ↑CHOP → Apoptosis	*In Vitro*: Inhibited cell viability, induced apoptosis, mitochondrial depolarization, ROS generation, and reduced GSH levels, while promoting cell migration inhibition, autophagy, and activation of ERS signaling. *In Vivo*: Combined with Thapsigargin, significantly inhibited tumor growth in xenograft models	Ma et al. (2017)
Celecoxib	NSAID	Celecoxib → ↑GRP78, ↑CHOP, ↑caspase-4 → Apoptosis	*In Vitro*: Induces apoptosis in A549 and H460 lung cancer cells, with a higher apoptosis rate observed in A549 cells, involving caspase-4 activation and ER stress markers like GADD34 and CHOP.	Kim et al. (2017)
Phenethyl isothiocyanate (PEITC)	Experimental	PEITC → PERK↑ → eIF2α↑ → CHOP↑ → Noxa↑ → Mcl-1↓ → ↑ Apoptosis PEITC + Gefitinib → Increased ER stress → Enhanced apoptosis	*In Vitro*: PEITC+Gefitinib inhibited cell growth and induced apoptosis, decreasing the antiapoptotic protein Mcl-1 via a proteasomal degradation mechanism involving the ER stress pathway. *In Vivo*: The PEITC-Gefitinib combination significantly inhibited NCI-H1299 xenograft tumor growth, without causing significant toxicity	Zhang et al. (2020c)
Rafoxanide	Anthelmintic	Rafoxanide → GRP78, p-eIF2α, ATF6, XBP1s → Apoptosis/Autophagy	*In Vitro*: Decreases cell growth, migration, and invasion, induces apoptosis and cell cycle arrest, and activates ER stress and autophagy. *In Vivo*: Suppresses tumor growth in xenograft mouse models without significant side effects on liver or kidney function	Hu et al. (2023)
Pemetrexed (PEM)	Anticancer	PEM → ↓ PERK → ↓p-eIF2α → ↓ATF4 → ↓CHOP, ↓ GRP78 → ↓ ER Stress → Partial restoration of protein synthesis under low glucose	*In Vitro*: Reduces protein synthesis under low glucose conditions by inhibiting UPR signaling, including the PERK, ATF4, and CHOP pathways. *In Vivo*: Alleviates glucose deprivation-induced protein synthesis repression by reducing UPR activation in A549 cells	Piecyk et al. (2021)
Glycyrrhetinic acid (GA)	Experimental	GA → ↑ Unfolded Proteins → ↑ Bip → ↑ PERK → ↓ Protein Synthesis → G1 Cell Cycle Arrest	*In Vitro*: Inhibits cell proliferation, induces G0/G1 phase cell cycle arrest, and upregulates ER stress-related proteins without inducing apoptosis	Zhu et al. (2015)
Catechin-7-O-xyloside (C7Ox)	Experimental	C7Ox → ↑ ER stress → ↑ CHOP → ↑ Caspase-6 activation → ↑ Apoptosis	*In Vitro*: C7Ox induces cell death, apoptosis, and necrosis via mitochondrial damage, caspase activation, and ER stress, with CHOP playing a key role in mediating the cytotoxic effects. *In Vivo*: C7Ox inhibits tumor growth in a lung carcinoma xenograft model	Yoon et al. (2014)
Chaetocin	Experimental	Chaetocin → Inhibits SUV39H1 → ↑ ER stress → ↑ ATF3 and CHOP → ↑ DR5 → Apoptosis	*In Vitro*: Inhibits NSCLC cell survival by inducing apoptosis	Liu et al. (2015)
H1 (a bromized derivative of tetrandrine)	Experimental	H1 → ↑ CHOP → ↑ DR5 → ↑ Caspase-8, -9, -3, and PARP cleavage → Apoptosis	*In Vitro*: Induces caspase-dependent apoptosis, upregulates DR5 and CHOP, downregulates c-FLIP, triggers ER stress, and promotes autophagy in human NSCLC cells	Lin et al. (2016)
Epimedokoreanin B (EKB)	Experimental	EKB → ↑ER Stress → ↑UPR (GRP78, IRE1α, PERK, ATF6, ATF4, p-eIF2α, GADD153) → Paraptosis + Autophagosome Accumulation	*In Vitro*: EKB treatment decreased cell survival and migration in NSCLC cells and triggered paraptosis	Zheng et al. (2022)
Afzelin	Experimental	Afzelin → ↓ NQO2 → ER stress ↑ → (↑ p-PERK, ↑ p-eIF2α, ↑ GRP78, ↑ CHOP) → ↑ Immunogenic cell death	*In Vitro*: Inhibits lung cancer cell proliferation, promotes apoptosis, induces ER stress, and enhances ICD by increasing ER stress markers and ICD markers (ATP, CRT, HMGB1), with NQO2 as a key target. *In Vivo*: NQO2 overexpression reverses Afzelin-induced effects on cell proliferation, apoptosis, and ER stress in A549 and H1299 cells	Xia et al. (2023)
Fascaplysin	Experimental	Fascaplysin ↑ → ATF4 ↑ → HSPA5 and CHOP ↑ → ER stress ↑ Fascaplysin ↑ → ATF4 ↑ → SLC7A11 ↑ → Ferroptosis ↑	*In Vitro*: Induced apoptosis and ferroptosis by increasing ROS, upregulating PDL1 expression, and modulating ferroptosis-related proteins such as GPX4 and FTH1. *In Vivo*: Enhanced the anti-tumor efficacy of anti-PD1 immunotherapy by promoting PDL1 expression, increasing immune infiltration (CD4^+^ and CD8^+^ T cells), and inducing ferroptosis in a mouse model of NSCLC.	Luo and Xu (2022)
UA232 (a derivative of Ursolic Acid)	Experimental	UA232 → ↑ PERK/eIF2α → ↑ CHOP → Apoptosis	*In Vitro*: Inhibits cell proliferation, induces G0/G1 cell cycle arrest, and promotes apoptosis. *In Vivo*: ER stress inhibition partially reverses UA232-induced apoptosis	Gou et al. (2020b)
Deoxypodophyllotoxin (DPT)	Experimental	DPT → ↑GRP78, CHOP, DR5, DR4 → ↑ER stress → Apoptosis	*In Vitro*: Inhibits EGFR and MET kinase activity in an ATP-competitive manner, suppresses cell growth, induces apoptosis and causes G2/M cell cycle arrest	Kim et al. (2021)
Nelfinavir Chloroquine	Nelfinavir (HIV protease inhibitor) Chloroquine (anti-malarial)	Nelfinavir → ↑ ER stress (ATF3, p-eIF2α) → Proteotoxicity → Apoptosis Chloroquine → ↑ ER stress → Inhibits autophagy → Apoptosis Nelfinavir + Chloroquine → ↑↑ ER stress → ↑ Proteotoxicity → ↑ Apoptosis	*In Vitro*: Nelfinavir and chloroquine synergistically inhibit cell proliferation, enhance proteotoxicity via ubiquitin accumulation, induce apoptosis via PARP cleavage, and increase ER stress without affecting autophagy. *In Vivo*: The combination significantly suppresses NSCLC xenograft tumor growth in mice, enhances apoptosis via PARP cleavage, and induces ER stress	Lopiccolo et al. (2021)
Fish Oil (FO) (Omega-3 Fatty Acids) Selenium (Se)	Experimental	FO + Se → ↑ CHOP → Apoptosis ↑ FO + Se → ↓ GRP78 → ER Stress ↑ → Sensitivity to Gefitinib ↑ FO + Se → ↓ β-Catenin and COX-2 → ER Stress ↑ → Less Resistance	*In Vitro*: FO+Se reversed enhanced gefitinib-induced apoptosis, and restored drug sensitivity	Liao et al. (2020)
ZX-29 (novel ALK inhibitor)	Experimental	ZX-29 → ↑ ER stress → ↑ (PERK, p-PERK, IRE1a, p-IRE1a, GRP78, CHOP) → Apoptosis ↑	*In Vitro*: Inhibited anaplastic lymphoma kinase (ALK) activity, reduced NCI-H2228 cell viability, suppressed proliferation by inducing G1-phase arrest, promoted apoptosis and induced protective autophagy. *In Vivo*: Suppressed ALK and tumor growth in mice without noticeable toxicity	Gou et al. (2020a)
ART (Artemisinin) SOMCL-14-221 (221, artemisinin derivative)	Artemisin (anti-malarial)	ART or 221 → ↑ PERK → ↑ eIF2α phosphorylation → ↑ ATF4 → ↑ CHOP → Apoptosis	*In Vitro*: ART and 221 inhibit cell proliferation, migration, and colony formation, induce G0/G1 cell cycle arrest, and activate ER stress pathways, with 221 showing stronger effects. *In Vivo*: ART and 221 suppress tumor growth in A549 xenograft models without significant toxicity, demonstrating comparable efficacy to 5-fluorouracil	Xiao et al. (2020)
Aspirin Celecoxib	NSAIDs	Aspirin → GRP78 inhibition → ER stress → caspase-7 and caspase-12 activation → apoptosis Celecoxib: No direct effect on ER stress	*In Vitro*: Aspirin+Celecoxib inhibited cell proliferation, induced apoptosis, arrested the cell cycle at the G0/G1 phase, and suppressed cell migration by targeting GRP78, enhancing ROS generation, and inhibiting the ERK-MAPK pathway. *In Vivo*: The combination therapy suppressed tumor growth, showing superior efficacy compared to monotherapy with either aspirin or celecoxib	Zhang et al. (2020d)
Total Ginsenosides Extract (TGS)	Experimental	TGS → ↑ ER stress (ATF4, CHOP, BIP) → ↑ Autophagy → Autophagic Cell Death	*In Vitro*: Induces autophagic cell death in cells by increasing autophagy flux and activating ER stress via AKT1-mTOR inhibition	Zhao et al. (2019)
Oxymatrine	Experimental	Oxymatrine → ↑ ER stress → ↑ Caspase-12 → Apoptosis	*In Vitro*: Reduced cell viability, induced apoptosis, caused G0/G1 cell cycle arrest, disrupted cytoskeletal organization, and inhibited EMT by upregulating E-cadherin and downregulating N-cadherin	Izdebska et al. (2019)
Penfluridol	Experimental	Penfluridol → ↑ ER Stress → ↑ UPR (GRP78, PERK, IRE1α, CHOP) → ↑ Autophagosome Accumulation → ↓ ATP Energy → Nonapoptotic Cell Death	*In Vitro*: Inhibits cell proliferation and migration, induces autophagosome accumulation without apoptosis, and suppresses growth via ER stress-mediated UPR and p38 MAPK activation, leading to ATP depletion. *In Vivo*: Suppresses tumor growth and metastasis in mouse model by inducing autophagosome accumulation	Hung et al. (2019)
P-NFs Nano-filaments	Experimental	P-NFs → PERK, IRE1 → JNK Phosphorylation → CHOP expression → Bax ↑, Bcl-2 ↓ → Apoptosis ↑	*In Vitro*: Enhance cytotoxicity, apoptosis, and ER stress in cells more effectively than free Paclitaxel, with a stronger impact on Bax/Bcl-2 regulation	(He et al., 2019)
Homoisoflavanone-1	Experimental	Homoisoflavanone-1 → ↑ PERK → ↑ ATF4 → ↑ GADD34	*In Vitro*: Inhibits proliferation, induces G2/M arrest, triggers apoptosis via mitochondrial and ER stress pathways, and regulates key cell cycle and apoptotic proteins in A549 cells	Ning et al. (2018)
SH-EAE (Scindapsus cf. hederaceus extract)	Experimental	SH-EAE → ↓ PERK and IRE-1α → ↓ ER stress tolerance → ↑ cancer cell vulnerability​	*In Vitro*: Reduced proliferation, migration, and invasion of cells, without inducing apoptosis or DNA damage. *In Vivo*: Suppressed angiogenesis in zebrafish models by reducing intersegmental vessel formation without affecting larval development	Chou et al. (2018)
OT52 (coumarin derivative)	Experimental	OT52 → ↑GRP78/Bip, ↑PERK, ↑p-eIF2α, ↑ATF4, ↑ATF6, ↑CHOP → ER stress	*In Vitro*: Induced cytostatic effects in cells without toxicity, leading to G1-phase arrest, cellular senescence, metabolic collapse, ER/Golgi stress, STAT3 inhibition, and suppression of spheroid formation, migration, and stemness. *In Vivo*: Exhibited no acute toxicity or cardiotoxicity in zebrafish embryos	Lee et al. (2018)
Doxorubicin (Dox)	Anticancer	Dox → ↑ ER Stress → ↑ UPR (↑p-eIF2α, ↑ GADD153) → Apoptosis	*In Vitro*: Doxorubicin induces apoptosis and UPR activation in NSCLC cells, with UPR preconditioning enhancing apoptosis via the eIF2α/mTOR pathway, while Tauroursodeoxycholate inhibits this effect	Zhao et al. (2016)
β-elemene (from Curcuma wenyujin)	Experimental	β-elemene → ↑ PERK/IRE1α/ATF6 → ↑ CHOP → ↓ Bcl-2 → Apoptosis → ↓ Tumor Growth	*In Vitro*: Inhibits proliferation, induces ROS generation, and triggers apoptosis via the PERK/IRE1α/ATF6-mediated ER stress pathway. *In Vivo*: Suppresses tumor growth in mice and enhances immune function	Liu et al. (2017)
Licochalcone A (LicA)	Experimental	LicA → ↑ p-EIF2α → ↑ ATF4 → ER stress → Apoptosis	*In Vitro*: Inhibits the proliferation by inducing G2/M cell cycle arrest, apoptosis, and ER stress in a dose-dependent manner. *In Vivo*: Exhibits antitumor effects in animal models, showing therapeutic potential against lung cancer	Qiu et al. (2017)
Emodin	Experimental	Emodin → ↑ER stress (GRP78, CHOP) → TRIB3/NF-κB activation → Apoptosis	*In Vitro*: Reduces cell viability and induces apoptosis via ER stress, with TRIB3/NF-κB signaling playing a key role. *In Vivo*: Suppresses tumor growth in nude mice by promoting ER stress-dependent apoptosis	Su et al. (2017)
Obovatol	Experimental	Obovatol → ↑ ER stress (↑ CHOP, IRE1α, ATF4, p-eIF2) → Apoptosis	*In Vitro*: Induces apoptosis by regulating apoptosis and ER stress-related proteins, with CHOP playing a key role in apoptosis induction	Kim et al. (2016c)
AHA (Mannose-binding lectin from Arisaema heterophyllum Blume)	Experimental	AHA → ↑ ER stress (↑ p-eIF2α, CHOP, IRE1α, p-JNK) → Apoptosis (↑ Bax/Bcl-2 ratio, caspase-9/3 activation, ↓ XIAP, ↓ PI3K/Akt) and Autophagy (↑ LC3-II, ↑ ATG7)	*In Vitro*: Inhibits cell proliferation, induces G1 phase arrest, apoptosis, autophagy, and ER stress, while modulating the PI3K/Akt pathway	Feng et al. (2016)
A17 (Curcumin analog) Curcumin	Experimental	A17 → ↑ GRP78 → ↑ XBP-1, ATF4 → ↑ CHOP → Caspase activation → Apoptosis	*In Vitro*: Shows stronger cytotoxicity than Curcumin, induces apoptosis, ER stress, and caspase activation, and apoptosis is suppressed by CHOP silencing	Ye et al. (2016)
Licochalcone A (LCA)	Experimental	LCA → ↑ BIP → ↑ CHOP → ↑ Apoptosis (caspase-3/7 activation)	*In Vitro*: Induces apoptosis, autophagy, and ER stress in a CHOP-dependent manner, while not affecting normal cells	Tang et al. (2016)
Ethanolic extract of Descurainia sophia seeds (EEDS)	Experimental	EEDS ⟶ ↑ CHOP ⟶ ↑ DR5/DR4 ⟶ TRAIL sensitivity ⟶ ↑ Apoptosis	*In Vitro*: Sensitized A549 lung cancer cells to TRAIL-induced apoptosis by upregulating TRAIL receptors, particularly DR5, in a CHOP-dependent manner	Park et al. (2016)
Podophyllotoxin analog (PA)	Experimental	PA → ↑ ER stress → ↑ CHOP, phospho-PERK, IRE1-α, phospho-JNK → Apoptosis	*In Vitro*: Induced cell death via microtubule disruption, G2/M cell cycle arrest, DNA damage, ER stress, and autophagic signaling	Choi et al. (2015)
Anacardic acid (AA)	Experimental	AA → IRE1α ↑ → UPR ↑ → ER stress ↑ → CHOP ↑, Caspase-12 cleavage ↑, Mitochondrial damage ↑ → Apoptosis ↑	*In Vitro*: AA induced cytotoxicity, Ca2+ mobilization, ER stress, autophagy, and mitochondrial damage in A549 cells, triggering cell death via the UPR and pro-apoptotic markers	Seong et al. (2014)
B82 (a monocarbonyl analog of Curcumin)	Experimental	B82 → ER stress ↑ (GRP78 ↑, ATF-4 ↑, XBP-1 ↑, CHOP ↑) → Caspase-3 activation ↑ → Apoptosis ↑ → Tumor growth ↓	*In Vitro*: Exhibited potent anti-tumor effects, inducing apoptosis and activating ER stress-mediated pathways. *In Vivo*: Inhibited tumor growth in H460 xenograft models, promoting ER stress-mediated apoptosis and reducing oncogene expression	Liu et al. (2013)
Luteolin	Experimental	Luteolin → ↑ ER stress → ↑ p-eIF2α → ↑ CHOP → Cell death	*In Vitro*: Induced apoptosis in NCI-H460 cells via both extrinsic and intrinsic pathways, involving caspase activation and ER stress, and also triggered autophagy as a death mechanism	Park et al. (2013)
PS-341 (Bortezomib)	Clinical use (multiple myeloma)	PS-341 → ER Stress ↑ → ATF4 ↑ → ATF3 and CHOP ↑ → DR5 ↑ → Apoptosis PS-341 → PKCδ ↑ → ER Stress ↑ → ATF4, ATF3, CHOP ↑ → DR5 ↑ → Apoptosis PS-341 → ERK/RSK2 ↑ → DR5 ↑ → Apoptosis	*In Vitro*: Induces apoptosis, with ERK/RSK2 and PKCd pathways playing key roles. *In Vivo*: Induces apoptosis in tumor models, with PKCδ and ERK/RSK2 regulating these processes	Xu et al. (2012)
Salermide	Experimental	Salermide → Sirt1/2 inhibition → ATF4↑ → ATF3↑ → CHOP↑ → DR5↑ → Apoptosis	*In Vitro*: Induces apoptosis in a concentration- and time-dependent manner, primarily via upregulation of DR5 via the ATF4-ATF3-CHOP axis	Liu et al. (2012)
Ardisiphenol D (Hendriks et al., 2024) Ardisiphenol E (Siegel et al., 2024)	Experimental	1 and 2 → ↑ ER stress → ↑ CHOP, GRP78, IRE1 → G1 Arrest and Apoptosis 1 and 2 → ↑ CHOP → Caspase-9, Caspase-3 activation → Apoptosis	*In Vitro*: Inhibit cell growth, induce ER stress, cause G1 cell cycle arrest, and trigger apoptosis in H1299 and A549 cells	Zhu et al. (2012)
(1E,4E)-1,5-bis(2,3-dimethoxyphenyl) penta-1,4-dien-3-one (compound 19)	Experimental	Compound 19 → ↑ GRP78 → ↑ ATF-4 and XBP-1 → ↑ CHOP → ↑ IRE1 → ↑ JNK → ↑ c-Jun → ↑ p53 → ↑ Caspase-3 and Caspase-9 → Apoptosis → ↓ Tumor Growth	*In Vitro*: Induces apoptosis in H460 cells. *In Vivo*: Inhibits H460 tumor growth in nude mice, with reductions in tumor volume	Wang et al. (2011)
Tanshinone IIA	Experimental	Tanshinone IIA Mechanism: → ↑ PERK → ↑ ATF4 → ↑ CHOP → ↑ DR5 → ↑ TRAIL sensitivity → Apoptosis Tanshinone IIA + TRAIL → ↑ DR5 → TRAIL sensitivity ↑ → Enhanced apoptosis	*In Vitro*: Anshinone IIA+TRAIL synergistically induces apoptosis in TRAIL-resistant NSCLC cells by upregulating DR5, activating the PERK/ATF4 pathway, and suppressing the STAT3/survivin axis	Kim et al. (2016b)

A significant number of agents predominantly activate the PERK pathway. These compounds induce phosphorylation of eIF2α, leading to the upregulation of ATF4 and CHOP. The resulting inhibition of global protein synthesis and promoting apoptotic signaling is a recurring theme. For example, the SERCA inhibitor thapsigargin depletes ER Ca^2+^ stores, provoking ER stress and robust activation of the PERK–eIF2α–ATF4–CHOP axis, thereby linking calcium homeostasis disruption to PERK-driven apoptosis. In several studies, enhanced PERK signaling correlates with increased sensitivity to apoptosis, while its attenuation has been linked to chemoresistance. Conversely, other agents preferentially stimulate the IRE1 branch of the UPR. IRE1 activation results in the splicing of XBP1 mRNA and can initiate downstream pro-apoptotic signals, such as JNK activation via the IRE1/TRAF2/ASK1 complex. The IRE1-mediated pathway is involved in apoptosis and regulating autophagy and other stress responses, indicating its multifaceted role in cell fate decisions under ER stress conditions.

A smaller subset of compounds exerts its effects mainly through the ATF6 branch. Activating ATF6 leads to increased transcription of ER chaperones like GRP78/BiP, which initially helps restore ER homeostasis. However, sustained ATF6 activation, particularly when coupled with signals from the PERK and IRE1 branches, can drive cells toward apoptosis. Thus, the ATF6 response is seen as a double-edged sword that, under severe or prolonged stress, contributes to cell death.

Notably, many agents induce a broad UPR activation, affecting all three branches simultaneously. This multi-pronged engagement produces a robust ER stress response that overwhelms the cell’s adaptive mechanisms and leads to enhanced apoptotic or non-apoptotic forms of cell death, such as paraptosis. Such observations have spurred interest in combination therapies that leverage the synergistic effects of targeting multiple UPR branches, thereby potentially overcoming the limitations of agents that modulate a single pathway.

The general conclusion is that diverse therapeutic agents can modulate the UPR by selectively or simultaneously targeting the PERK, IRE1, and ATF6 branches. This differential engagement underscores the complexity of ER stress signaling in cancer and highlights the therapeutic promise of finely tuning these pathways to induce cell death in resistant tumors.

### 3.3 Overcoming therapy resistance

Therapy resistance remains a significant hurdle in cancer treatment, often leading to relapse and limited patient survival. A growing body of research highlights the potential of modulating ER stress to overcome resistance, particularly by leveraging the UPR to drive cancer cells toward apoptosis. Numerous experimental and clinically approved therapeutic agents have been investigated for their ability to sensitize tumors to conventional treatments such as chemotherapy and radiation by engaging key UPR pathways.

The rationales for combination therapies stem from the synergistic enhancement of ER stress and apoptosis through multiple signaling pathways, such as ROS induction, calcium dysregulation, PERK/IRE1α signaling, and autophagy modulation. These combinations not only amplify the cytotoxicity of chemotherapeutic agents and radiotherapy but also overcome key mechanisms of chemoresistance and radioresistance, such as cancer stem cell traits and pro-survival ER stress pathways. By integrating different pathways that target ER stress and cell death mechanisms, these therapies offer a more comprehensive approach to tackling NSCLC, particularly in cases with drug resistance.

#### 3.3.1 Enhancing chemosensitivity

Several compounds have demonstrated efficacy in enhancing chemosensitivity through ER stress modulation (summarized in Supplementary Table S3). Curcumin has been shown to induce ER stress by upregulating CHOP and ATF6, thereby increasing apoptosis and sensitizing cells to Cisplatin. When combined with Cisplatin, Curcumin amplifies ER stress, leading to significantly higher apoptotic rates and reduced cell viability (Wang et al., 2022).

Similarly, Diphyllin enhances Cisplatin-induced apoptosis by inhibiting SERCA2, disrupting ER calcium homeostasis, and triggering mitochondrial calcium overload, ultimately increasing cytochrome C release and ROS production (Xu Z. et al., 2024).

Urtica dioica extract enhances ER stress-induced apoptosis by upregulating GADD153, DR5, and caspase-8, leading to increased cell death. Combined with Cisplatin, it exerts a synergistic anti-proliferative effect, further amplifying apoptosis (D’Abrosca et al., 2019).

Regorafenib, a multi-kinase inhibitor, has also emerged as a potent ER stress inducer, increasing NOX5-mediated ROS production and activating the PERK/eIF2α/ATF4 pathway to enhance apoptosis. When used alongside Cisplatin, regorafenib exhibits a synergistic effect, further elevating ROS levels and apoptotic signaling (Sui et al., 2023).

Comparable mechanisms are observed with Polydatin, which similarly engages NOX5 and ROS pathways, reinforcing ER stress-driven cell death (Wu S. et al., 2024). Other natural compounds, such as Icariside II (Tang et al., 2022), Delicaflavone (Wang et al., 2020), and Dihydroartemisinin (Ni et al., 2024), have been shown to upregulate key UPR components in combination with Cisplatin, promoting increased tumor cell apoptosis and reduced tumor growth.

Additionally, the combination of Honokiol and Paclitaxel has synergistic effects in inducing paraptosis in NSCLC cells by promoting proteasomal inhibition, ER stress, and mitochondrial dysfunction, ultimately leading to significant tumor growth delay in xenograft models (Li et al., 2021).

#### 3.3.2 Reversing chemoresistance

In some cancers, intrinsic resistance mechanisms involve the upregulation of pro-survival ER stress pathways, which mitigate the cytotoxic effects of chemotherapeutic agents (Supplementary Table S3). For example, Astragaloside IV counteracts Cisplatin resistance by downregulating GRP78 and PERK, restoring chemosensitivity (Lai et al., 2020). Selenium Yeast (Se-Y) combined Fish Oil (FO) also reverses Cisplatin resistance by activating AMPK, suppressing GRP78, promoting CHOP-mediated apoptosis, and reducing cancer stem cell traits (Lai et al., 2022). Similarly, Paris Saponin II enhances Cisplatin cytotoxicity by intensifying ER stress and inducing paraptosis—a non-apoptotic form of programmed cell death linked to excessive ER swelling (Man et al., 2020).

#### 3.3.3 ER stress as a radiosensitization strategy

In addition to chemotherapy, ER stress modulation has been explored to enhance radiotherapy’s effectiveness.

Chalcomoracin, an experimental agent, induces ER dilation and upregulates Bip and CHOP, leading to increased paraptosis. When combined with radiation therapy, Chalcomoracin further amplifies ER stress, enhancing tumor radiosensitivity and inhibiting tumor growth (Zhang SR. et al., 2020; Li et al., 2025). Similar effects are observed with β-apopicropodophyllin, which augments radiation-induced ER stress by activating BiP, PDI, phospho-eIF2α, and ATF4, leading to enhanced apoptosis and delayed tumor progression (Kim et al., 2019).

PPARγ agonists, such as PPZ023 and Ciglitazone, also potentiate radiation therapy by promoting ER stress-mediated apoptosis. However, their effects appear context-dependent, as they enhance radiosensitivity in NSCLC cells but show limited efficacy in radioresistant cell populations. These findings highlight the need for a more nuanced understanding of ER stress modulation in radiation therapy (Kim et al., 2020).

In summary, targeting ER stress pathways offers a promising strategy for overcoming therapy resistance in cancer. Therapeutic agents that modulate UPR components can enhance chemosensitivity, reverse chemoresistance, and improve radiosensitivity by tipping the balance from adaptive survival to apoptotic cell death. Future research should focus on optimizing combination strategies, identifying biomarkers for patient selection, and minimizing potential toxicities associated with prolonged ER stress activation.

### 3.4 Other combinational therapies

Beyond traditional chemotherapy and radiotherapy, various experimental and clinically approved agents have been explored in combination to enhance ER stress-driven apoptosis and overcome cancer resistance. These approaches target different cellular stress pathways, including proteotoxic stress, oxidative stress, calcium homeostasis, and autophagy regulation, offering new therapeutic possibilities for hard-to-treat cancers.

Maintaining protein homeostasis is critical for cancer cell survival, making the proteostasis network a prime target for therapy. The combination of Carfilzomib and Suberanilohydroxamic Acid, a histone deacetylase.

HDAC6 inhibitor, exemplifies this approach. Carfilzomib prevents the degradation of misfolded proteins, leading to ER stress activation, while Suberanilohydroxamic Acid blocks Aggresome formation, further exacerbating proteotoxic stress. Together, these agents create a synergistic accumulation of misfolded proteins, increase ROS, and intensify ER stress responses through ATF4, GRP78/BiP, and CHOP activation, ultimately driving apoptosis (Hanke et al., 2016).

Several drug combinations leverage oxidative stress to amplify ER stress and promote cancer cell death. For instance, Celastrol and Afatinib synergistically increase ROS levels, inducing ER stress and triggering paraptosis, a non-apoptotic form of programmed cell death (Dai et al., 2021).

When combined with tyrosine kinase inhibitors (TKIs) such as Erlotinib and Gefitinib, Shikonin enhances ROS-mediated ER stress through increased eIF2α and ATF4 expression, leading to cytotoxicity (Li et al., 2018).

Similarly, a combination of Phenethyl Isothiocyanate and Gefitinib enhances ER stress-induced apoptosis by upregulating PERK-eIF2α-CHOP signaling, increasing pro-apoptotic Noxa and downregulating the survival protein Mcl-1, thereby amplifying cancer cell death (Zhang Q. et al., 2020).

Autophagy modulation is another promising strategy. While Sorafenib induces autophagy flux and ER stress, FTY720 (Fingolimod) suppresses autophagy, further intensifying ER stress when combined with sorafenib or lapatinib. This combination enhances cytotoxicity, providing a potential treatment avenue for resistant tumors (Ota et al., 2019).

The ER calcium homeostasis is tightly linked to stress responses and cell survival. Thapsigargin, a SERCA inhibitor, depletes ER calcium stores, leading to increased ER stress. When combined with Calmodulin antagonists such as Trifluoperazine and ophiobolin A, which further promote calcium leakage, this combination induces severe ER stress, pushing cancer cells toward apoptosis (Linxweiler et al., 2013). Similarly, Ardisiphenol D and E activate CHOP and Bip while inhibiting Skp2, leading to G1 arrest and caspase-dependent apoptosis (Zhu et al., 2012).

Several compounds have been investigated for their ability to enhance the effectiveness of pro-apoptotic agents. Decursin increases ROS levels and upregulates ATF4, PERK, and CHOP, thereby enhancing the expression of DR5 and sensitizing cancer cells to TRAIL-induced apoptosis (Kim J. et al., 2016). A similar effect is observed with Tanshinone IIA, which promotes DR5 expression via PERK/ATF4/CHOP activation, increasing cancer cell susceptibility to TRAIL-mediated apoptosis (Kim EO. et al., 2016).

Interestingly, drugs initially developed for non-cancer indications have demonstrated potent anticancer effects through ER stress modulation. Nelfinavir, an HIV protease inhibitor, induces proteotoxicity by upregulating ATF3 and p-eIF2α, leading to apoptosis. When combined with chloroquine, an autophagy inhibitor that further enhances ER stress, the two drugs synergistically increase proteotoxic stress, making this combination a potential repurposing strategy for cancer therapy (Lopiccolo et al., 2021).

Nutritional components such as Se-Y and Fish Oil have also been explored for their ability to modulate ER stress and improve therapy outcomes. These compounds activate AMPK, upregulate CHOP, and downregulate GRP78, leading to increased apoptosis and reduced cancer stem cell traits (Lai et al., 2022). Additionally, FO and Se have been shown to reduce β-catenin and COX-2 levels, increasing sensitivity to gefitinib and decreasing therapy resistance (Liao et al., 2020).

By targeting diverse cellular stress mechanisms, these combination therapies offer promising new avenues for enhancing ER stress-induced cytotoxicity in cancer. Whether through proteostasis disruption, oxidative stress amplification, calcium homeostasis dysregulation, or autophagy modulation, these strategies provide a framework for overcoming therapy resistance.

## 4 Predictive biomarkers for ER-stress–targeting strategies

A practical stratification framework for ER-stress–modulating strategies in NSCLC separates baseline markers of UPR tone from on-treatment pharmacodynamic markers. Baseline markers such as GRP78/BiP and the XBP1 splicing ratio estimate how much a tumor is already relying on chaperone buffering or IRE1 signaling; this helps choose which arm to target (PERK/IRE1/ATF6, ROS amplification, Ca^2+^ handling, or proteostasis). Pharmacodynamic markers such as CHOP induction, p-eIF2α/ATF4 activation, and DR5 upregulation verify on-target UPR engagement and align with apoptotic response across many agents summarized in this review. In multiple models, agents that raise proteotoxic or oxidative load co-vary with upregulation of CHOP and DR5, whereas GRP78 is context-dependent: inhibition or down-modulation often tracks with sensitization to EGFR TKIs or Cisplatin (Lai et al., 2020; Liao et al., 2020; Zhang X. et al., 2020; Kim et al., 2017; Liu et al., 2013).

GRP78/BiP is the canonical ATF6-responsive chaperone; high baseline levels suggest an adaptive UPR buffering misfolded proteins. Baseline high GRP78 may mark tumors dependent on chaperone capacity; combinations that lower GRP78 (such as aspirin-based regimens, fish-oil/selenium, Astragaloside IV) frequently restore sensitivity or heighten apoptosis (Lai et al., 2020; Liao et al., 2020; Xiao et al., 2020).

Second, on-treatment change in GRP78 is informative; a decrease from baseline can serve as a pharmacodynamic marker of effective UPR disruption, whereas transient increases can reflect stress sensing rather than commitment to apoptosis (Kim et al., 2017; Liu et al., 2013).

XBP1 mRNA splicing (XBP1s) is a measurable indicator of IRE1 RNase activity and is repeatedly detected alongside response to IRE1-engaging regimens. Here, the baseline ratio of XBP1s to XBP1u is a reasonable enrichment marker for IRE1-axis targeting, while an on-treatment rise in XBP1s serves as a pharmacodynamic indicator that the IRE1 branch is engaged (Yuan et al., 2023; Xiao et al., 2012; Man et al., 2020; Kim et al., 2017; Liu et al., 2013; Wang et al., 2011; Qian et al., 2023).

Across proteasome stressors, ROS amplifiers, and Ca^2+^ disruptors, the most consistent pro-apoptotic pharmacodynamic marker is coordinated induction of p-eIF2α and ATF4 with CHOP. CHOP often couples to DR5 upregulation and TRAIL sensitivity (Sui et al., 2023; Dai et al., 2018; Ni et al., 2024; Kim EO. et al., 2016; Yoon et al., 2014; Liu et al., 2015; Gou et al., 2020a; Park et al., 2016; Xu et al., 2012; Liu et al., 2012). Incorporating p-eIF2α, ATF4, CHOP, and optionally DR5 as a pharmacodynamic marker allows you to confirm apoptotic UPR commitment and to predict synergy with death-receptor strategies (such as TRAIL agonism) or proteasome-targeting agents.

Several additional indicators refine mechanism-specific choices. ATF6 activation (like GRP78 target induction) complements PERK/IRE1 indicators and helps distinguish adaptive from terminal UPR states (Yuan et al., 2023; Wang et al., 2019; Kim et al., 2017). Immunogenic cell death markers, like calreticulin exposure, ATP release, HMGB1, are especially useful when pairing ER-stress inducers with checkpoint blockade, as ICD upregulation suggests added immunogenic benefit (Yuan et al., 2023; Zheng et al., 2022). ROS/NOX5 signatures track benefit from ROS-amplifying regimens and Cisplatin combinations (Sui et al., 2023; Zhang J. et al., 2020; Zhao et al., 2022; Hu et al., 2020; Li et al., 2018; Wu S. et al., 2024). SEC62 reflects Ca^2+^ handling; high SEC62 marks sensitivity to Ca^2+^-disrupting ER stress (such as thapsigargin, calmodulin antagonists) (Linxweiler et al., 2013). Finally, polyubiquitinated protein accumulation serves as a pharmacodynamic indicator of proteostasis stress and paraptosis with CFZ±SAHA, 6-shogaol, or plumbagin (Hanke et al., 2016; Binoy et al., 2019; Nedungadi et al., 2018).

## 5 Challenges and opportunities

NSCLC remains a leading cause of cancer-related mortality globally, with therapy resistance posing a significant barrier to improving patient outcomes. Emerging evidence underscores ER stress and the UPR as central mediators of tumor survival, progression, and resistance to conventional therapies. The UPR’s dual role—balancing adaptive pro-survival signaling under transient stress and triggering apoptosis under sustained stress—highlights its complexity as a therapeutic target. Key UPR pathways (PERK, IRE1α, ATF6) are dysregulated in NSCLC, enabling cancer cells to evade apoptosis while adapting to hypoxia, nutrient deprivation, and therapeutic insults (Salvagno et al., 2022; Zhang W. et al., 2024).

Therapeutic strategies targeting ER stress leverage diverse mechanisms, including ROS induction, calcium dysregulation, and proteasome inhibition, to shift the UPR balance toward cell death. Agents such as proteasome inhibitors, SERCA inhibitors, and natural compounds demonstrate preclinical efficacy by exacerbating ER stress, activating CHOP-driven apoptosis, or inducing non-apoptotic death pathways like paraptosis.

These approaches also activate immunogenic ICD, improving the efficacy of cancer immunotherapy by boosting anti-tumor immune responses. This activation helps to convert tumors with low immunogenicity into ones with high immunogenicity, making them more recognizable and targetable by the immune system (Arimoto et al., 2024; Wang ZB. et al., 2024).

Combination therapies hold particular promise. By integrating ER stress inducers with chemotherapy, radiotherapy, or targeted agents, resistance mechanisms can be circumvented through synergistic stress overload. For example, Cisplatin resistance is reversed by agents like Diphyllin or Astragaloside IV, which disrupt calcium homeostasis or downregulate pro-survival chaperones. Similarly, radiation efficacy is bolstered by compounds such as Chalcomoracin, which amplifies ER stress-mediated paraptosis.

The current evidence on therapeutic agents modulating ER stress highlights several experimental and clinical agents that could be repurposed to improve the treatment of NSCLC. By leveraging existing drugs used for various conditions, such as cancer, infections, and chronic diseases, we can enhance the therapeutic arsenal against NSCLC. For instance, the HIV protease inhibitor nelfinavir induces ER proteotoxic stress and, particularly in combination with chloroquine, augments apoptosis via ATF3 and p-eIF2α activation. Similarly, the kinase inhibitor regorafenib increases NOX5-mediated ROS and activates PERK/eIF2α/ATF4 signaling to enhance cisplatin cytotoxicity in NSCLC models.

Many agents used in cancer treatment, like Cisplatin, Pemetrexed, and Paclitaxel, are already integral to NSCLC therapy. Combinations with novel agents (e.g., Curcumin, Regorafenib, or Carfilzomib) could improve efficacy by enhancing the immune response or overcoming drug resistance. For example, combining Regorafenib with Cisplatin might increase tumor targeting while minimizing adverse effects.

Drugs initially designed for infections, such as Nifuroxazide or Dihydroartemisinin, could show synergistic effects with existing NSCLC treatments. Their repurposing in combination with chemotherapy or immunotherapy may enhance tumor cell killing or immune modulation. For instance, Nelfinavir and Chloroquine could improve the immune response and reduce tumor growth when used alongside radiation or chemotherapy. We can consider these drugs as combination therapies, targeting both NSCLC and the comorbidities like existing infections.

Moreover, drugs like Celecoxib and Aspirin have proven anti-inflammatory properties and can be repurposed in NSCLC for their potential to reduce tumor-associated inflammation, thereby enhancing treatment outcomes. Thus, we can consider Celecoxib and Aspirin as preferred analgesics in NSCLC patients.

Despite these advances and future opportunities, challenges persist. The UPR’s context-dependent outputs—pro-survival versus pro-death—necessitate precise modulation (Lundstrom, 2023) to avoid inadvertently promoting tumor resilience. A significant gap exists between preclinical research and clinical application. Most of the agents being reviewed here are still undergoing experimental studies (Ren et al., 2025; Zhang et al., 2025), and few validated clinical results can guarantee their safety, efficacy, and long-term benefits in NSCLC patients. Moreover, several translational barriers continue to hinder clinical progress. First, there is a lack of standardized and reliable clinical tools for detecting and monitoring ER stress responses in patients, which limits accurate assessment of therapeutic effects. Second, the absence of multicenter clinical validation and large-scale external datasets prevents robust confirmation of preclinical findings. Third, the inherent heterogeneity of tumor cells and their dynamic interactions with the surrounding immune and stromal microenvironment complicate the translation of ER stress-targeting strategies from controlled laboratory settings to diverse clinical contexts. Fourth, ethical and data-governance challenges—including privacy protection, informed consent, and the risk of genetic discrimination—must be addressed before implementing ER stress–based personalized treatments. Finally, a deeper mechanistic understanding of how ER stress pathways influence immune modulation and tumor behavior is still lacking, underscoring the need for comprehensive functional and longitudinal studies. The complex biology of cancer and individual patient variability means that these drugs must be tested in carefully designed human trials to assess their therapeutic potential and any possible adverse effects or toxicities in the context of NSCLC treatment (Marfatiya et al., 2025).

## 6 Conclusion

In conclusion, ER stress and the UPR play a pivotal yet complex role in NSCLC progression and therapy resistance. While the UPR initially acts as a pro-survival mechanism under transient stress, sustained activation shifts its role toward promoting apoptosis, presenting a therapeutic opportunity. Current strategies targeting ER stress—such as ROS induction, calcium dysregulation, and proteasome inhibition—demonstrate preclinical efficacy in overcoming resistance to chemotherapy, radiotherapy, and targeted therapies. Combination therapies, particularly those integrating ER stress inducers with conventional treatments, show synergistic potential by overwhelming cancer cell adaptive responses and inducing immunogenic cell death. However, the dual nature of the UPR necessitates precise modulation to avoid inadvertently enhancing tumor resilience. Challenges in translating these findings into clinical practice include understanding context-dependent UPR outcomes, optimizing drug combinations, and addressing tumor heterogeneity. Future research should prioritize biomarker discovery, personalized therapeutic approaches, and rigorous clinical trials to validate the safety and efficacy of ER stress-targeting agents. By bridging these gaps, exploiting ER stress pathways could transform NSCLC treatment paradigms, offering hope for improved survival in this challenging malignancy.
