# Supplementary material for: Endoplasmic reticulum stress in non-small cell lung cancer: a review of therapeutic agents, mechanistic insights, and implications for therapy
Source: Front Cell Dev Biol. 2025 Dec 16;13:1693023. doi: 10.3389/fcell.2025.1693023 (PMC12748239; doi:10.3389/fcell.2025.1693023)
Supplement: Supplementary file 1 [file Table1.docx]

**Table S1**. *Summary of therapeutic agents, their impact on ROS/ER stress pathways, and anti-cancer effects*

| Therapeutic Agent | Drug Category | Axis | Outcomes | Refs |
| --- | --- | --- | --- | --- |
| Marsdenia tenacissima extract (MTE) | Anticancer | - MTE → ↑ ER stress markers (ATF6, GRP78, ATF4, XBP1s, CHOP) → Triggers immunogenic cell death - MTE → ↑ ROS and ↓ MMP → ER stress | ***In Vitro***: Increase ER stress, leading to ICD. ***In Vivo***: Increases ER stress markers and ICD markers (ATP, HMGB1). | (1) |
| Nifuroxazide (NFZ) | Antibiotic | - NFZ → ROS ↑ → Ca2+ ↑ → PERK ↑ → ATF4 ↑ → CHOP ↑ → ↑ Apoptosis | ***In Vitro***: Induces ROS. Activates the PERK/ATF4/CHOP ERS pathway to trigger apoptosis. Suppresses JAK2/STAT3 signaling. These effects are reversible with PERK pathway inhibition. | (2) |
| Regorafenib | Anticancer | - Regorafenib → ↑NOX5 → ↑ROS → ↑p-eIF2α/ATF4 → ↑ Apoptosis - Cisplatin + Regorafenib → Synergistic ↑ROS → ↑p-eIF2α/ATF4 → ↑↑ Apoptosis | ***In Vitro***: Inhibiting cell proliferation, reducing colony formation, and inducing apoptosis via caspase-dependent mechanisms. Increasing intracellular ROS levels, leading to ER stress activation, and enhancing Cisplatin cytotoxicity synergistically. ***In Vivo***: Regorafenib+Cisplatin suppressed tumor growth, accompanied by increased ER stress and MAPK pathway activation. No significant toxicity in major organs. | (3) |
| Carfilzomib (CFZ) Suberanilohydroxamic Acid (SAHA) | Anticancer | - CFZ → Proteasome inhibition → ↑ Misfolded proteins → ↑ ER stress (ATF4, GRP78, CHOP) - SAHA → HDAC6 inhibition → Aggresome formation blocked → ↑ Misfolded proteins → ↑ ER stress (ATF4, GRP78, CHOP) - CFZ + SAHA → Proteasome inhibition + HDAC6 inhibition → ↑↑ misfolded protein + ROS ↑↑ → ↑↑ ER stress (ATF4, GRP78, CHOP) → Apoptosis ↑ | ***In Vitro***: CFZ + SAHA synergistically suppressed cell proliferation, with enhanced cytotoxicity and apoptosis. Proteasome inhibition, and induced ER stress. Increased ROS. | (4) |
| Curcumol | Experimental | - Curcumol → NQO2 inhibition → ROS increase → ER stress activation → CHOP upregulation → DR5 increase → TRAIL sensitization → Apoptosis | ***In Vitro***: Curcumol synergistically enhanced TRAIL-induced apoptosis. Curcumol elevate ROS levels, which triggered ER stress. ***In Vivo***: Curcumol/TRAIL co-treatment significantly reduced tumor growth in A549 xenograft models without causing toxicity. | (5) |
| Celastrol | Experimental | - Afatinib + Celastrol → ↑ ROS → ↑ ER stress → ↑ UPR → ↑ Paraptosis → ↑ Cell death | ***In Vitro***: Celastrol+Afatinib synergistically inhibited the survival of Afatinib-resistant cells by inducing paraptosis. The process was driven by intracellular ROS accumulation and mitochondrial Ca²⁺ overload. ***In Vivo***: Afatinib+Celastrol suppressed tumor growth in mouse model. The combination was more effective than either drug alone. | (6) |
| Celastrol | Experimental | - Celastrol → ↑ ROS → ER Stress (↑ ATF4, ↑ P-eIF2α) → Apoptosis - Celastrol → ↑ ROS → ↓ STAT3 Pathway → Apoptosis | ***In Vitro***: Suppressed cell viability, migration, and invasion and induced apoptosis by promoting ROS generation, inhibited the IL-6/STAT3 signaling pathway, reducing p-STAT3 nuclear translocation. ***In Vivo***: Reduced tumor volume and weight without significant toxicity to vital organs in xenograft mouse model. | (7) |
| Shikonin AZD9291 (Osimertinib) | Experimental | - Shikonin / AZD9291 → ↑ ROS → ↑ ER stress (ATF-4, p-eIF2α) → ↑ Apoptosis - Shikonin + AZD9291 → ↑↑ ROS → ↑↑ ER stress → ↑↑ Apoptosis | ***In Vitro***: Shikonin enhanced AZD9291 cytotoxicity by reducing survival, suppressing colony formation, and inducing apoptosis, driven by ROS generation. | (8) |
| Curcumin | Experimental | - Curcumin → ROS ↑ → ER stress ↑ (↑ GRP78, IRE1α, CHOP, PERK) → DNA damage ↑ → G2/M arrest ↑ → Apoptosis ↑ | ***In Vitro***: Inhibits cell proliferation and colony formation while inducing G2/M cell cycle arrest. Promotes apoptosis via the mitochondrial pathway. Induces ROS accumulation, leading to oxidative stress and activation of ER stress-related proteins. ***In Vivo***: Curcumin suppresses tumor growth. | (9) |
| Butein | Experimental | - Butein → Increase in ROS → ER Stress Activation (PERK, IRE1α, CHOP, eIF2α) → Apoptosis | ***In Vitro***: Reduced cell viability, inhibited cell adhesion, migration, and invasion, increased intracellular ROS levels and activated ER stress pathways. ***In Vivo***: Inhibited tumor xenograft growth in mice. | (10) |
| Shikonin | Experimental | - Shikonin → ROS → ↑ eIF2α, ↑ ATF4 → ER stress - Erlotinib/Gefitinib + Shikonin → ROS → ↑ eIF2α, ↑ ATF4 → ER stress | ***In Vitro***: Enhanced the antiproliferative effects of erlotinib and gefitinib, inducing apoptosis, ROS generation, and ER stress. ***In Vivo***: Enhanced the tumor-suppressive effects of erlotinib and gefitinib, significantly reducing tumor volume when combined, whereas low doses of the drugs alone had minimal impact. | (11) |
| WZ35 (a mono-carbonyl analog of Curcumin) | Experimental | - WZ35 induces ROS → ROS activates ER stress pathway (UPR) → Phosphorylation of eIF2α → Increased ATF4 and CHOP → Apoptosis | ***In Vitro***: Reduced cell viability, compared to gefitinib and erlotinib, induced apoptosis, increased G2/M cell cycle arrest. The apoptotic effects were mediated by ROS generation. ***In Vivo***: Inhibited tumor growth in xenograft mouse model. Treatment reduced tumor volume and weight without affecting body weight. | (12) |
| Decursin TRAIL (TNF-related apoptosis-inducing ligand) | Experimental | - Decursin → ROS ↑ → ATF4/PERK ↑ → CHOP ↑ → DR5 ↑ → TRAIL sensitivity ↑ → Apoptosis ↑ - TRAIL + Decursin → DR5 activation → Caspase activation → Apoptosis ↑ | ***In Vitro***: Decursin+TRAIL synergistically reduced the viability of TRAIL-resistant cells by inducing apoptosis via activation of the extrinsic pathway. | (13) |
| Curcumin | Experimental | - Curcumin → ↑ ROS → ↑ ER stress proteins (GADD153, GRP78) → ↑ Apoptosis - Curcumin → ↑ Ca²⁺ release from ER → Mitochondrial damage (ΔΨm loss) → Apoptosis | ***In Vitro***: Induced G2/M phase arrest and apoptosis. ***In Vivo***: Treatment in xenograft models showed reduced tumor growth. | (14) |
| Fucoidan | Experimental | - Fucoidan → ↑ ROS → ↑ ER stress - Fucoidan → ↑ GRP78 → ↑ PERK → ↑ eIF2α → ↑ ATF4 → ↑ CHOP → ↑ Apoptosis - Fucoidan → ↓ AKT/ERK → ↓ Cancer cell viability - Fucoidan → ↑ p21 → Cell cycle arrest → ↓ Tumor growth | ***In Vivo***: Reduces tumor growth in LLC1 mice. Mice pretreated exhibited smaller tumor volumes with no toxicity. ***In Vitro***: Inhibited proliferation and viability by inducing ER stress, increasing GRP78, ATF4, and CHOP expression while suppressing AKT and ERK phosphorylation. It promoted apoptosis via ROS generation and upregulation of caspase 3 and PARP. | (15) |

**Table S2**. *Summary of therapeutic agents modulating ER stress via calcium metabolism and proteasome function*

| Therapeutic Agent | Drug Category | Axis | Outcomes | Refs |
| --- | --- | --- | --- | --- |
| **Agents mainly modulating the calcium metabolism** | | | | |
| Diphyllin | Experimental | Diphyllin ↓ SERCA2 → ER Ca²⁺ depletion → ER stress ↑ (↑ GRP78, ATF4, DDIT3, p-PERK, p-eIF2α) → Apoptosis ↑ Diphyllin ↓ SERCA2 → Mitochondrial Ca²⁺ overload → Mitochondrial dysfunction ↑ → Cytochrome c release ↑ → Caspase activation ↑ → Apoptosis ↑ Diphyllin → ER stress ↑ → Enhances Cisplatin-induced apoptosis | ***In Vitro***: Inhibited cell growth by inducing G0/G1 cell cycle arrest and apoptosis, impaired migration and invasion while disrupting ER calcium homeostasis by inhibiting SERCA2. This led to ER calcium depletion, cytosolic and mitochondrial calcium overload, ROS accumulation, mitochondrial dysfunction, and cytochrome c release, ultimately triggering apoptosis. ***In Vivo***: In xenograft models, Diphyllin suppressed tumor growth in mice without causing notable toxicity. | (16) |
| Nifuroxazide (NFZ) | Antibiotic | NFZ → ROS ↑ → Ca2+ ↑ → PERK ↑ → ATF4 ↑ → CHOP ↑ → Apoptosis | ***In Vitro***: NFZ reduced cell viability, induced apoptosis, and increased ROS and Ca²⁺ levels. It activated the ER stress. NFZ also inhibited JAK2/STAT3 phosphorylation. | (2) |
| Plumbagin (PLB) | Experimental | PLB → ↑ ROS → ↑ Ca²⁺ → ↑ (CHOP, ATF4, p-eIF2α) → Apoptosis | ***In Vitro***: plumbagin (PLB) inhibited cell growth and induced apoptosis by reducing mitochondrial membrane potential and increasing oxidative stress and Ca²⁺ levels. ***In Vivo***: PLB suppressed tumor growth and improved survival in mouse models without affecting body weight. Additionally, PLB significantly reduced tumor burden. | (17) |
| Tanshinone IIA (Tan IIA) | Experimental | Tan IIA → ↑Ca²⁺ → ↑ER stress → ↑JNK → ↓NFAT2 → ↓c-Myc | ***In Vitro***: Tan IIA inhibited the growth and induced apoptosis. The drug also reduced colony formation and increased apoptosis rates. ***In Vivo***, Tan IIA suppressed tumor growth in both] xenograft and LLC mouse models, without affecting body weight. Combining Tan IIA with anti-PD-1 therapy enhanced its anti-tumor efficacy, reducing tumor volume and proliferation markers in the mouse models. | (18) |
| LW-213 (a derivative of wogonin) | Experimental | LW-213 ⟶ NPC1 inhibition ⟶ Cholesterol depletion in ER ⟶ Ca²⁺ release ⟶ FAM134B activation ⟶ Reticulophagy ⟶ ER stress ⟶ Apoptosis | ***In Vitro***: LW-213 induced apoptosis. Additionally, LW-213 altered cholesterol metabolism by inhibiting the LDLR-PCSK9 pathway. Lysosomal damage was observed. | (19) |
| Recombinant scyreprocin (rScyreprocin) | Experimental | rScyreprocin → ROS ↑ → ER stress ↑ → Ca²⁺ release ↑ → Mitochondrial dysfunction ↑ → Apoptosis ↑​ | ***In Vitro***: rScyreprocin inhibited proliferation, migration, and colony formation of cancer cells, while showing no toxicity to non-cancer cells. It induced apoptosis and membrane disruption, leading to cancer cell death. ***In Vivo***: rScyreprocin suppressed tumor growth in mice model, inducing significant necrosis and apoptosis in tumors without affecting body weight. It also reduced Ki-67 and CD31 areas, indicating decreased tumor proliferation and angiogenesis. | (20) |
| Honokiol | Experimental | Paclitaxel + Honokiol → Proteasomal inhibition → ER stress ↑ → UPR ↑ → ER dilation ↑ → Paraptosis ↑ Paclitaxel + Honokiol → Ca²⁺ homeostasis disruption → Mitochondrial Ca²⁺ overload → Mitochondrial dysfunction ↑ → Paraptosis ↑ | ***In Vitro***: Paclitaxel and honokiol synergistically induce paraptosis, with a significant effect on paclitaxel-resistant cells. ***In Vivo***: In a mouse H1299 xenograft model, the combination suppressed tumor growth and induced paraptosis in tumor cells, with minimal side effects, particularly no significant body weight loss. | (21) |
| Thapsigargin Calmodulin Antagonists: Trifluoperazine (TFP), Ophiobolin A | Experimental  Antipsychotic Experimental | Thapsigargin → Inhibits SERCA → ER Ca²⁺ depletion → ER stress ↑ TFP / Ophiobolin A → Inhibit calmodulin → ER Ca²⁺ leakage → ER stress ↑ Thapsigargin + TFP/Ophiobolin → Stronger ER stress ↑↑ | ***In Vitro***: SEC62 silencing or treatment with calmodulin antagonists disrupts ER Ca²⁺ homeostasis, reduces cell migration, and increases sensitivity to thapsigargin-induced ER stress. ***In Vivo***: High SEC62 levels in NSCLC tissues correlate with poor survival, especially in SCC, suggesting its potential as a prognostic marker. | (22) |
| Curcumin | Experimental | Curcumin → ↑ ROS → ↑ ER stress proteins (GADD153, GRP78) → ↑ Apoptosis Curcumin → ↑ Ca²⁺ release from ER → Mitochondrial damage (ΔΨm loss) → Apoptosis | ***In Vitro***: Curcumin induced G2/M cell cycle arrest, apoptosis via caspase activation, ROS generation, mitochondrial dysfunction, ER stress, and modulation of apoptosis-related proteins in NCI-H460 lung cancer cells. | (14) |
| B63 (a synthetic Curcumin analog) | Experimental | B63 → ER Calcium Depletion → UPR Activation (GRP78, ATF4, XBP-1, CHOP) → Apoptosis (Caspase-3, Caspase-9) → Tumor Growth Inhibition | ***In Vitro***: B63 exhibited higher cellular uptake and stability compared to Curcumin, inducing potent apoptosis via ER stress, with significant effects on ER calcium depletion. ***In Vivo***: B63 significantly inhibited tumor growth in mice, showing reduction in tumor volume and weight. | (23) |
| **Agents mainly modulating the proteasome function** | | | | |
| Carfilzomib (CFZ) Suberanilohydroxamic Acid (SAHA) | Anticancer | CFZ → Proteasome inhibition → ↑ Misfolded proteins → ↑ ER stress (ATF4, GRP78/GRP78, CHOP) SAHA → HDAC6 inhibition → Aggresome formation blocked → ↑ Misfolded proteins → ↑ ER stress (ATF4, GRP78/GRP78, CHOP) CFZ + SAHA → Proteasome inhibition + HDAC6 inhibition → ↑↑ misfolded protein + ROS ↑↑ → ↑↑ ER stress (ATF4, GRP78/GRP78, CHOP) → Apoptosis ↑ | ***In Vitro***: Synergistic increased cell death and apoptosis | (4) |
| Honokiol Paclitaxel | Experimental | Paclitaxel + Honokiol → Proteasomal inhibition → ER stress ↑ → UPR ↑ → ER dilation ↑ → Paraptosis ↑ Paclitaxel + Honokiol → Ca²⁺ homeostasis disruption → Mitochondrial Ca²⁺ overload → Mitochondrial dysfunction ↑ → Paraptosis ↑ | ***In Vitro***: Synergistic killing of NSCLC cells via paraptosis induction. ***In Vivo***: Growth delay in xenograft tumors. | (21) |
| Plumbagin | Experimental | Plumbagin ⟶ Proteasome inhibition ⟶ ER stress ↑ (GRP78, CHOP accumulation) Plumbagin ⟶ Sulfhydryl disruption ⟶ ER stress ↑ ER stress ↑ ⟶ Cytoplasmic vacuolation ⟶ Paraptosis | ***In Vitro***: Induces paraptosis and cell cycle arrest | (24) |
| 6-Shogaol (6S) (derived from Ginger) | Experimental | 6S ⟶ Proteasome inhibition ⟶ ↑ Polyubiquitinated proteins ⟶ ↑ ER stress (↑ GRP78, ↑ CHOP) ⟶ ER-derived vacuolation ⟶ Paraptosis (caspase-independent) | ***In Vitro***: Decreases cell viability, colony formation, causes G1 phase arrest, and induces ER vacuolation then paraptosis | (25) |

**Table S3**. *Summary of Therapeutic Agents Enhancing Chemosensitivity and Reversing Chemoresistance via ER Stress Modulation*

| Therapeutic Agents | Drug Category | Cell lines | Axis | Therapy Outcomes | Refs |
| --- | --- | --- | --- | --- | --- |
| Curcumin | Experimental | Parental: A549, H1299. Cisplatin-resistant: A549/DDP, H1299/DDP | - Curcumin → ↑ ER stress (CHOP, ATF6) → ↑ Apoptosis → ↑ Cisplatin sensitivity. - Cisplatin → ↑ ER stress → ↑ Apoptosis - Curcumin + Cisplatin → ↑↑ ER stress → ↑↑ Apoptosis → Enhanced Cisplatin sensitivity. | Increases the sensitivity of drug-resistant cells to Cisplatin by decreasing cell viability, promoting apoptosis, and regulating ER stress-related proteins. | (26) |
| Diphyllin | Experimental | A549, PC9, Lewis lung carcinoma  BEAS-2B, 8505C, MCF-7, A498, AGS, HCT116. | - Diphyllin → Inhibits SERCA2 → ↓ ER Ca²⁺ → ↑ cytosolic Ca²⁺ → ↑ Mitochondrial Ca²⁺ overload → ROS ↑ → ↑ Cytochrome C release → ↑ Apoptosis - Cisplatin → DNA damage → ↑ Apoptosis - Diphyllin → ER stress ↑ → Enhances Cisplatin-induced apoptosis | Sensitizes NSCLC cells to chemotherapy by inhibiting SERCA2, disrupting Ca2+ signaling, and enhancing the effects of Cisplatin, resulting in increased cell apoptosis and reduced tumor growth. | (16) |
| Regorafenib | Anticancer | H1299, PC-9 | - Regorafenib → ↑NOX5 → ↑ROS → ↑p-eIF2α/ATF4 → ↑ Apoptosis - Cisplatin + Regorafenib → Synergistic ↑ROS → ↑p-eIF2α/ATF4 → ↑↑ Apoptosis. | Sensitized cancer cells to Cisplatin by increasing ROS levels, activating ER stress and MAPK pathways. | (3) |
| Icariside II | Experimental | A549, H1299, Lewis lung carcinoma Cisplatin-resistant A549/DDP | - Icariside II → ↑PERK, ↑p-PERK, ↑IRE1α, ↑p-IRE1α, ↓ATF6 → ↑eIF2α, ↑p-eIF2α, ↑ATF4, ↑CHOP → ↑ Apoptosis - Icariside II + Cisplatin → ↑PERK, ↑p-PERK, ↑IRE1α, ↑p-IRE1α, ↓ATF6 → ↑eIF2α, ↑p-eIF2α, ↑ATF4, ↑CHOP → ↑↑ Apoptosis | The combination of IS and Cisplatin enhances chemotherapy sensitization by increasing cell apoptosis, promoting ER stress, and suppressing tumor growth and angiogenesis, with no significant toxicity observed ***In Vivo***. | (27) |
| Delicaflavone | Experimental | A549, PC9,  Cisplatin-resistant: A549/DDP PC9/DDP | - Delicaflavone/ Cisplatin → ↑GRP78, ↑CHOP → Apoptosis | Enhanced Cisplatin sensitivity in resistant cancer cells by inhibiting proliferation, migration, and invasion while promoting apoptosis via the ER stress pathway. | (28) |
| Astragaloside IV | Experimental | A549, H1299,  Cisplatin-resistant: A549Cis, H1299Cis. | - Cisplatin → ↑ GRP78, ↑ PERK → Chemoresistance - AS-IV → ↓ GRP78, ↓ PERK → Chemosensitivity | Sensitized Cisplatin-resistant cancer cells by enhancing Cisplatin’s anti-tumor effects via suppression of ER stress and autophagy. | (29) |
| Dihydroartemisinin (DHA) | Anti-malarial | A549, H460 | - DHA → ROS → ER Stress (↑ p-eIF2α, ↑ ATF4) → ↑ Apoptosis - Cisplatin → ROS → ER Stress (↑ p-eIF2α, ↑ ATF4) → ↑ Apoptosis - DHA + Cisplatin → ROS → ER Stress (↑↑ p-eIF2α, ↑↑ ATF4) → ↑↑ Apoptosis | DHA+Cisplatin enhanced anti-NSCLC effects by promoting ROS accumulation, activating ER stress, and stimulating the JNK and p38 MAPK pathways. | (30) |
| Polydatin (PD) | Experimental | H1299, H460 | - PD → ↑NOX5 → ↑ROS → ↑ER stress (p-eIF2α, ATF4) → ↑ Apoptosis. - Cisplatin → ↑ROS → ↑ER stress (p-eIF2α, ATF4) → ↑ Apoptosis. - PD + Cisplatin → ↑NOX5 + ↑ROS → ↑ER stress (p-eIF2α, ATF4) + ↑JNK/p38 MAPK → ↑↑ Apoptosis | Enhances the cytotoxic effects of Cisplatin and carboplatin in cancer cells by promoting ROS-mediated ER stress, JNK and p38 MAPK signaling, and NOX5 activation, leading to synergistic tumor suppression. | (31) |
| Selenium yeast (Se-Y) + fish oil (FO) | Dietary Supplements | A549 | - Se-Y + FO → AMPK activation → CHOP ↑, GRP78 ↓ → Apoptosis ↑, CSC traits ↓, Cisplatin resistance reversed | Se-Y+FO enhanced Cisplatin sensitivity in, reversing Cisplatin resistance by increasing apoptosis and suppressing CSC traits, including ABCG2 and EMT markers. | (32) |
| Paris Saponin II (PSII) | Experimental | NCI-H460, NCI-H520 | - PSII → ER Stress ↑ (PERK, eIF2α, ATF4, CHOP, IRE1α, XBP1) → Paraptosis ↑ - PSII + Cisplatin → ER Stress ↑↑ → Paraptosis ↑↑ → Cytotoxicity ↑↑ | Sensitized cancer cells to Cisplatin by inducing paraptosis, resulting in increased cytotoxicity and synergistic effects when combined with Cisplatin. | (33) |
| Kushenol Z (KZ) | Experimental | A549, NCI-H226 | - KZ ↓ cAMP-PDE → ↑ cAMP → ↑ PKA → ↑ CHOP, caspase-7, caspase-12 → ↑ ER stress → Apoptosis - KZ ↓ Akt → ↑ PRAS40 → ↓ mTOR → ↑ ER stress → Apoptosis | Sensitizes cancer cells to Cisplatin by inhibiting cell proliferation, inducing apoptosis via mitochondrial and ER stress pathways, and downregulating the mTOR pathway. | (34) |
| Urtica dioica (UD) extract | Experimental | H460, H1299, A549, H322, Beas2B, WI38 | - UD → ↑GADD153 → ↑DR5 → ↑Caspase-8 → ↑ tBid → ↑Apoptosis - UD → ↑GADD153 → ↑ER stress → ↑Apoptosis - UD + Cisplatin → ↑Apoptosis (synergistic effect) | Enhanced Cisplatin sensitivity in cancer cells, improving apoptotic rates and inhibiting cell proliferation. | (35) |

# References

1. Yuan Y, Guo Y, Guo ZW, Hao HF, Jiao YN, Deng XX, et al. Marsdenia tenacissima extract induces endoplasmic reticulum stress-associated immunogenic cell death in non-small cell lung cancer cells through targeting AXL. J Ethnopharmacol. 2023 Oct;314:116620.

2. Li D, Liu L, Li F, Ma C, Ge K. Nifuroxazide induces the apoptosis of human non‑small cell lung cancer cells through the endoplasmic reticulum stress PERK signaling pathway. Oncol Lett. 2023 Jun;25(6):248.

3. Sui H, Xiao S, Jiang S, Wu S, Lin H, Cheng L, et al. Regorafenib induces NOX5-mediated endoplasmic reticulum stress and potentiates the anti-tumor activity of cisplatin in non-small cell lung cancer cells. Neoplasia. 2023 May;39:100897.

4. Hanke NT, Garland LL, Baker AF. Carfilzomib combined with suberanilohydroxamic acid (SAHA) synergistically promotes endoplasmic reticulum stress in non-small cell lung cancer cell lines. J Cancer Res Clin Oncol. 2016 Mar;142(3):549–60.

5. Zhang J, Zhou Y, Li N, Liu WT, Liang JZ, Sun Y, et al. Curcumol Overcomes TRAIL Resistance of Non-Small Cell Lung Cancer by Targeting NRH:Quinone Oxidoreductase 2 (NQO2). Adv Sci (Weinheim, Baden-Wurttemberg, Ger. 2020 Nov;7(22):2002306.

6. Dai CH, Zhu LR, Wang Y, Tang XP, Du YJ, Chen YC, et al. Celastrol acts synergistically with afatinib to suppress non-small cell lung cancer cell proliferation by inducing paraptosis. J Cell Physiol. 2021 Jun;236(6):4538–54.

7. Zhao Z, Wang Y, Gong Y, Wang X, Zhang L, Zhao H, et al. Celastrol elicits antitumor effects by inhibiting the STAT3 pathway through ROS accumulation in non-small cell lung cancer. J Transl Med. 2022 Nov;20(1):525.

8. Hu X, Zhang ZY, Wu LW, Zeng LH, Chen H, Zhu HJ, et al. A natural anthraquinone derivative shikonin synergizes with AZD9291 against wtEGFR NSCLC cells through reactive oxygen species-mediated endoplasmic reticulum stress. Phytomedicine. 2020 Mar;68:153189.

9. Wang C, Song X, Shang M, Zou W, Zhang M, Wei H, et al. Curcumin exerts cytotoxicity dependent on reactive oxygen species accumulation in non-small-cell lung cancer cells. Future Oncol. 2019 Apr;15(11):1243–53.

10. Di S, Fan C, Ma Z, Li M, Guo K, Han D, et al. PERK/eIF-2α/CHOP Pathway Dependent ROS Generation Mediates Butein-induced Non-small-cell Lung Cancer Apoptosis and G2/M Phase Arrest. Int J Biol Sci. 2019;15(8):1637–53.

11. Li YL, Hu X, Li QY, Wang F, Zhang B, Ding K, et al. Shikonin sensitizes wild‑type EGFR NSCLC cells to erlotinib and gefitinib therapy. Mol Med Rep. 2018 Oct;18(4):3882–90.

12. Dai X, Zhang J, Guo G, Cai Y, Cui R, Yin C, et al. A mono-carbonyl analog of curcumin induces apoptosis in drug-resistant EGFR-mutant lung cancer through the generation of oxidative stress and mitochondrial dysfunction. Cancer Manag Res. 2018;10:3069–82.

13. Kim J, Yun M, Kim EO, Jung DB, Won G, Kim B, et al. Decursin enhances TRAIL-induced apoptosis through oxidative stress mediated- endoplasmic reticulum stress signalling in non-small cell lung cancers. Br J Pharmacol. 2016 Mar;173(6):1033–44.

14. Wu SH, Hang LW, Yang JS, Chen HY, Lin HY, Chiang JH, et al. Curcumin induces apoptosis in human non-small cell lung cancer NCI-H460 cells through ER stress and caspase cascade- and mitochondria-dependent pathways. Anticancer Res. 2010 Jun;30(6):2125–33.

15. Hsu HY, Lin TY, Lu MK, Leng PJ, Tsao SM, Wu YC. Fucoidan induces Toll-like receptor 4-regulated reactive oxygen species and promotes endoplasmic reticulum stress-mediated apoptosis in lung cancer. Sci Rep. 2017 Mar;7:44990.

16. Xu Z, Shi Y, Zhu L, Luo J, Hu Q, Jiang S, et al. Novel SERCA2 inhibitor Diphyllin displays anti-tumor effect in non-small cell lung cancer by promoting endoplasmic reticulum stress and mitochondrial dysfunction. Cancer Lett. 2024 Aug;598:217075.

17. Jiang ZB, Xu C, Wang W, Zhang YZ, Huang JM, Xie YJ, et al. Plumbagin suppresses non-small cell lung cancer progression through downregulating ARF1 and by elevating CD8(+) T cells. Pharmacol Res. 2021 Jul;169:105656.

18. Zhang YZ, Lai HL, Huang C, Jiang ZB, Yan HX, Wang XR, et al. Tanshinone IIA induces ER stress and JNK activation to inhibit tumor growth and enhance anti-PD-1 immunotherapy in non-small cell lung cancer. Phytomedicine. 2024 Jun;128:155431.

19. Wang H, Jiang Y, Zhu M, Li H, Chen H, Wang H, et al. LW-213, a derivative of wogonin, triggers reticulophagy-mediated cell death in NSCLC via lysosomal damage combined with NPC1 inhibition. Phytomedicine. 2024 Nov;134:155958.

20. Yang Y, Chen HY, Hao H, Wang KJ. The Anticancer Activity Conferred by the Mud Crab Antimicrobial Peptide Scyreprocin through Apoptosis and Membrane Disruption. Int J Mol Sci. 2022 May;23(10).

21. Li XQ, Ren J, Wang Y, Su JY, Zhu YM, Chen CG, et al. Synergistic killing effect of paclitaxel and honokiol in non-small cell lung cancer cells through paraptosis induction. Cell Oncol (Dordrecht, Netherlands). 2021 Feb;44(1):135–50.

22. Linxweiler M, Schorr S, Schäuble N, Jung M, Linxweiler J, Langer F, et al. Targeting cell migration and the endoplasmic reticulum stress response with calmodulin antagonists: a clinically tested small molecule phenocopy of SEC62 gene silencing in human tumor cells. BMC Cancer. 2013 Dec;13:574.

23. Xiao J, Wang Y, Peng J, Guo L, Hu J, Cao M, et al. A synthetic compound, 1,5-bis(2-methoxyphenyl)penta-1,4-dien-3-one (B63), induces apoptosis and activates endoplasmic reticulum stress in non-small cell lung cancer cells. Int J cancer. 2012 Sep;131(6):1455–65.

24. Binoy A, Nedungadi D, Katiyar N, Bose C, Shankarappa SA, Nair BG, et al. Plumbagin induces paraptosis in cancer cells by disrupting the sulfhydryl homeostasis and proteasomal function. Chem Biol Interact. 2019 Sep;310:108733.

25. Nedungadi D, Binoy A, Pandurangan N, Pal S, Nair BG, Mishra N. 6-Shogaol induces caspase-independent paraptosis in cancer cells via proteasomal inhibition. Exp Cell Res. 2018 Mar;364(2):243–51.

26. Wang L, Hu R, Dai A. Curcumin Increased the Sensitivity of Non-Small-Cell Lung Cancer to Cisplatin through the Endoplasmic Reticulum Stress Pathway. Evid Based Complement Alternat Med. 2022;2022:6886366.

27. Tang Z, Du W, Xu F, Sun X, Chen W, Cui J, et al. Icariside II enhances cisplatin-induced apoptosis by promoting endoplasmic reticulum stress signalling in non-small cell lung cancer cells. Int J Biol Sci. 2022;18(5):2060–74.

28. Wang X, Chen B, Xu D, Li Z, Sui Y, Lin X. Delicaflavone Reverses Cisplatin Resistance via Endoplasmic Reticulum Stress Signaling Pathway in Non-Small Cell Lung Cancer Cells. Onco Targets Ther. 2020;13:10315–22.

29. Lai ST, Wang Y, Peng F. Astragaloside IV sensitizes non-small cell lung cancer cells to cisplatin by suppressing endoplasmic reticulum stress and autophagy. J Thorac Dis. 2020 Jul;12(7):3715–24.

30. Ni L, Zhu X, Zhao Q, Shen Y, Tao L, Zhang J, et al. Dihydroartemisinin, a potential PTGS1 inhibitor, potentiated cisplatin-induced cell death in non-small cell lung cancer through activating ROS-mediated multiple signaling pathways. Neoplasia. 2024 May;51:100991.

31. Wu S, Zhao Q, Liu S, Kuang J, Zhang J, Onga A, et al. Polydatin, a potential NOX5 agonist, synergistically enhances antitumor activity of cisplatin by stimulating oxidative stress in non‑small cell lung cancer. Int J Oncol. 2024 Aug;65(2).

32. Lai IC, Liao CH, Hu MH, Chang CL, Lai GM, Chiou TJ, et al. Selenium Yeast and Fish Oil Combination Diminishes Cancer Stem Cell Traits and Reverses Cisplatin Resistance in A549 Sphere Cells. Nutrients. 2022 Aug;14(15).

33. Man S, Lv P, Cui J, Liu F, Peng L, Ma L, et al. Paris saponin II-induced paraptosis-associated cell death increased the sensitivity of cisplatin. Toxicol Appl Pharmacol. 2020 Nov;406:115206.

34. Chen H, Yang J, Hao J, Lv Y, Chen L, Lin Q, et al. A Novel Flavonoid Kushenol Z from Sophora flavescens Mediates mTOR Pathway by Inhibiting Phosphodiesterase and Akt Activity to Induce Apoptosis in Non-Small-Cell Lung Cancer Cells. Molecules. 2019 Dec;24(24).

35. D’Abrosca B, Ciaramella V, Graziani V, Papaccio F, Della Corte CM, Potenza N, et al. Urtica dioica L. inhibits proliferation and enhances cisplatin cytotoxicity in NSCLC cells via Endoplasmic Reticulum-stress mediated apoptosis. Sci Rep. 2019 Mar;9(1):4986.
